# Genetic Transfer in Action: Uncovering DNA Flow in an Extremophilic Microbial Community

**DOI:** 10.1111/1462-2920.70048

**Published:** 2025-02-03

**Authors:** Julia Van Etten, Timothy G. Stephens, Debashish Bhattacharya

**Affiliations:** ^1^ Department of Biochemistry and Microbiology, Rutgers The State University of New Jersey New Brunswick New Jersey USA

**Keywords:** community ecology, extremophiles, horizontal gene transfer, k‐mer analysis, metagenomics, mobile genetic elements

## Abstract

Horizontal genetic transfer (HGT) is a significant driver of genomic novelty in all domains of life. HGT has been investigated in many studies however, the focus has been on conspicuous protein‐coding DNA transfers that often prove to be adaptive in recipient organisms and are therefore fixed longer‐term in lineages. These results comprise a subclass of HGTs and do not represent exhaustive (coding and non‐coding) DNA transfer and its impact on ecology. Uncovering exhaustive HGT can provide key insights into the connectivity of genomes in communities and how these transfers may occur. In this study, we use the term frequency‐inverse document frequency (TF‐IDF) technique, that has been used successfully to mine DNA transfers within real and simulated high‐quality prokaryote genomes, to search for exhaustive HGTs within an extremophilic microbial community. We establish a pipeline for validating transfers identified using this approach. We find that most DNA transfers are within‐domain and involve non‐coding DNA. A relatively high proportion of the predicted protein‐coding HGTs appear to encode transposase activity, restriction‐modification system components, and biofilm formation functions. Our study demonstrates the utility of the TF‐IDF approach for HGT detection and provides insights into the mechanisms of recent DNA transfer.

## Graphical abstract

Different taxa from all domains of life sharing the same environment are connected by DNA transfers that have occurred between them. Whereas most studies appraising horizontal genetic transfer (HGT) events employ methods that only identify protein‐coding genes, here we apply a gene‐agnostic method to metagenomic data for the first time.

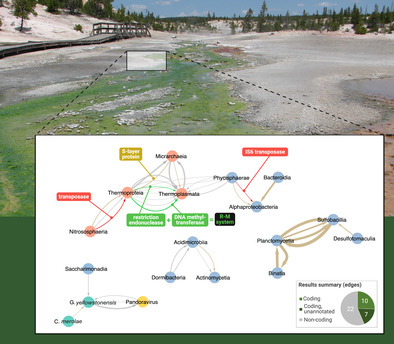

## 1 Introduction

Horizontal genetic transfer (HGT) is a driver of genomic novelty in prokaryotes and more recently, its significance has become recognised in eukaryotes (Ochman, Lawrence, and Groisman 2000; Koonin, Makarova, and Aravind 2001; Dagan, Artzy‐Randrup, and Martin 2008; Corel et al. 2018; Soucy, Huang, and Gogarten 2015; Van Etten and Bhattacharya 2020). Due to the advent and increasing availability of next‐generation sequencing technologies, investigation of the presence and adaptive significance of HGT have become commonplace. However, these studies have primarily focused on transfers involving protein‐coding genes, rather than exhaustive DNA transfers (either coding or non‐coding), their frequency in nature, and potential transfer mechanisms (Skippington and Ragan 2011; Rossoni et al. 2019; Brockhurst et al. 2019; Sibbald et al. 2020; Pereira, Christin, and Dunning 2023). This is in part because many HGTs are ancient acquisitions that have become fixed due to their clear adaptive significance, with many examples existing of this phenomenon. It took decades before this process was widely accepted in eukaryotes with the strongest evidence coming from cases involving transfer of protein‐coding genes with adaptive functions, which established the important role of HGT in eukaryote evolution (Van Etten and Bhattacharya 2020; Keeling and Palmer 2008; Huang 2013; Schönknecht, Weber, and Lercher 2014; Husnik and McCutcheon 2018). Furthermore, and perhaps more significantly, traditional methods for detecting HGT are based on phylogenies of expressed, protein‐coding genes which disregard DNA transfers that may be recent (and not yet under transcriptional control), non‐coding, or degraded (relic DNA from an ancient, non‐coding or no longer coding transfers, or regions that have experienced different degrees of mosaicism or amelioration) (Chan et al. 2009; Oren et al. 2014; Cong, Chan, and Ragan 2016). Identifying these types of transfers can provide insights into the true frequency of HGT and its background level in communities, a fundamental concept that has never been actively investigated. Moreover, there have been very few studies specifically investigating putative HGT donors, often due to the ancient nature of transfers of interest, incomplete sampling of the biosphere, and promiscuity of certain genes that obscures provenance (Lloyd et al. 2018; Lynch and Neufeld 2015; Kloub et al. 2021). Thus, there is much to be uncovered by investigating HGT in new and different ways.

To this end, metagenomics has become a useful and widespread tool for investigating community composition, organismal function and abundance, and ecology in a given environment (Taş et al. 2021). However, methods for HGT mining (especially those not focused on protein‐coding genes) have rarely been applied to this type of data (Douglas and Langille 2019), and pipelines and best practices for investigating genetic transfer across metagenome‐characterised microbial community data have not yet been established. Here, we utilise the term frequency‐inverse document frequency (TF‐IDF) statistic (implemented in the program, “tf‐idf” (Cong, Chan, and Ragan 2016)) to identify cases of DNA transfer within an environmental metagenomic dataset from Lemonade Creek, Yellowstone National Park (YNP). Our work provides proof‐of‐concept that: (1) DNA transfer can be studied at the community level, and (2) alignment‐free methods developed for high‐quality genome data can be successfully applied to less‐complete and lower coverage environmental genomic data. TF‐IDF compares the frequency of words within a document to their frequency in many documents within a “corpus”. If a particular word is rare in a given document or document set (term frequency statistic) but statistically much more frequently used within another group of documents within the broader corpus (inverse document frequency statistic), hypotheses can be formed about the relevance of that word (Wu et al. 2008; Qaiser and Ali 2018). This logic can be used to generate hypotheses when applied to genomic data, wherein genomes are analogous to documents and *k*‐mers (strings of nucleotides) derived from these genomes are analogous to words within those documents. Thus, if we compare a large number of genomes or metagenomes and find that a particular string of nucleotides is rare within the genomes of pre‐defined taxonomic group A (e.g., phylum, class, order), but is common within another pre‐defined taxonomic group B, we can hypothesize that the sequence was transferred from host group B to recipient group A (Cong, Chan, and Ragan 2016; Bernard et al. 2019). This method performs as well or better than other alignment‐free HGT detection methods, is effective for detecting recent transfers due to its use of *k*‐mers that mine HGTs at the nucleic acid level (rather than at the amino acid level, which is better for uncovering ancient transfers, but limits the results to protein‐coding regions), and is computationally quicker (Chan et al. 2009; Cong, Chan, and Ragan 2016; Bernard et al. 2019).

In this study, we applied the tf‐idf program to a large metagenomic dataset generated from an extremophilic microbial community collected from YNP (Stephens et al. 2024; Felipe Benites et al. 2024). We established a pipeline for filtering and validating results to make this method available to researchers looking to investigate what we term *genome connectivity*, or the inferred direct connections between two or more genomes in any environmental dataset that result from known DNA transfer events. Our results show that across the YNP geothermal community, non‐coding DNA transfers make up a majority of the genome‐to‐genome connections and most transfers are within‐domain acquisitions between bacterial or archaeal taxa. We also identified 10 transfers with high sequence identity to annotated proteins that encode a variety of functions, including transposase, endonuclease, and methyltransferase activity, and biofilm‐formation. These findings suggest that transposition and the restriction‐modification systems may facilitate HGT.

## 2 Experimental Procedures

### 2.1 Data Generation

The metagenome assembled genomes (MAGs) used for the tf‐idf analysis were generated by Stephens et al. (Stephens et al. 2024) and Benites et al. (Felipe Benites et al. 2024). A comprehensive description of sample collection, extraction, processing, and MAG construction and classification is provided in each publication as well as in Text S1.

### 2.2 k‐mer Analysis

A modified version of the tf‐idf program published by Cong et al. (Cong, Chan, and Ragan 2016) (https://github.com/TimothyStephens/TF‐IDF; modified to fix a memory leak when writing results) was used to identify putative DNA transfers between the two Cyanidiophyceae species (*Galdieria* and *Cyanidioschyzon*), the 174 non‐redundant prokaryotic MAGs, and the 25 non‐redundant viral MAGs. This Algal‐Prokaryotic‐Virus dataset is hereinafter referred to as the APV dataset. Based on the rigorous parameter testing already done by Cong et al., we chose to group the prokaryotic and viral MAGs by taxonomic class (Cong, Chan, and Ragan 2016); the two Cyanidiophyceae identified in the samples were kept as separate groups in the analysis. We ran tf‐idf using *k* = 25 and significance = 0.05, which were the optimal parameters identified by Cong et al. (Cong et al. 2017), however, we encourage readers trying similar analyses to use the supplemental information from Cong et al. (Cong et al. 2017) to determine if a *k* value of 25 is appropriate for their specific analysis.

### 2.3 HGT Filtering and Validation

**FIGURE 1:**
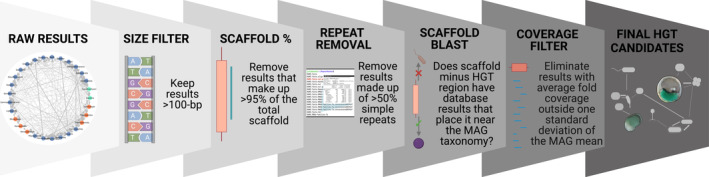
The filtering pipeline to validate putative HGTs identified using tf‐idf. All steps involve elimination of sub‐optimal or unverifiable results. See methods for an unabridged explanation of each of these steps. Made with Biorender.com.

The raw tf‐idf results (putative HGT events) were filtered to produce a conservative high‐quality subset that allowed for additional analysis of DNA transfers on a per‐event basis (Figure 1). The applied filtering steps are detailed below and in Figure 1, and a suite of accompanying summary statistics can be found in Table S1 (https://zenodo.org/records/10529606):

1. Putative HGT events < 100 bp in length were discarded. Longer HGTs are generally supported by more *k*‐mers and thus have higher intrinsic support, therefore, small HGT regions have less support and a higher chance of being false positives (additional information in Text S2). The 100‐bp threshold (~33 amino acids) was chosen to include small genes (which are generally assumed to be over 100 amino acids in length, but exceptions exist), fragments of coding regions, and non‐coding DNA, while also maintaining a minimum size which would allow for downstream BLAST or phylogenetic analysis.
2. Putative HGT events that covered > 95% of their respective scaffold length were discarded. Putative HGTs that cover almost the entire scaffold have little non‐HGT sequence that can be used to anchor it to the other scaffolds in the genome. That is, the non‐HGT region of a scaffold can be used to confirm its correct assignment to a MAG, reducing the likelihood of false positive HGT identifications due to chimeric MAGs. This is especially relevant to this particular dataset as the assemblies were built from short‐read sequencing data and thus, the scaffolds are relatively short and putative HGTs that take up whole scaffolds are challenging to validate.
3. HGTs on scaffolds that comprised > 50% simple repeats (identified using RepeatMasker (Smit, Hubley, and Green 2013–2015)) were removed. We also removed results from scaffolds with a high percentage (> 50%) of “N” (scaffold) characters. Simple repeats may result in false positive HGT identifications as they are often present in multiple unrelated species, not as a result of HGT. Highly scaffolded sequences also have a higher likelihood of producing false positive or uninformative HGT results. Therefore, HGTs on scaffold with a high proportion of simple repeats or “N” characters have a higher likelihood of being false positives and must be excluded from downstream analysis. The 50% cutoff was chosen based on the result that the highest simple repeat coverage below this cutoff was 37%, indicating that this filtering step eliminated sequences that were particularly simple repeat‐heavy, leaving just scaffolds that are predominantly non‐repetitive, and thus supports 50% as a reasonable threshold.
4. We queried the non‐HGT regions of each scaffold against the NCBI nr database using diamond blastx v2.1.2.156 (Buchfink, Reuter, and Drost 2021). If the top hit of each non‐HGT region was to an organism in the same class as the MAG that the scaffold was from, then the HGT was retained (we also retained results that had no blast hits as their provenance could not be accurately assessed). If the top hit was to another taxonomic group or an organism in the same group as the putative HGT donor, then this HGT was discarded because it had a phylogenetic signal that was inconsistent with the rest of the sequences in its MAG. This was used to check for obvious examples of a scaffold misattributed to a particular MAG wherein the HGT identified would be a false positive result.
5. Last, we compared the average fold coverage of the scaffolds containing putative HGTs with the average fold coverage of their respective MAGs. Read mapping was performed independently for each sample using bbmap.sh (Bushnell 2014). For the algal MAGs that were built from sequencing data from three different environments (soil, creek biofilm, and endolithic), we mapped the reads from a given environment against a reference composed of the prokaryotic and viral MAGs and the respective algal MAGs from the same environment. For example, the soil samples were mapped against a reference composed of the prokaryotic and viral MAGs, and the soil *Galdieria* and *Cyanidioschyzon* MAGs. Thus, if a putative HGT was identified within the soil *Galdieria* MAG, we only considered the average fold coverage values of the HGT in the soil samples. If a putative HGT was identified in a prokaryotic or viral MAG, then the average fold coverage values of the HGT across all 12 samples was considered. This approach was performed to prevent redundant algal sequences from different environments from sequestering reads during mapping, artificially reducing the average fold coverage of the algal MAG and causing any putative HGT scaffolds to have overly high coverage. For each MAG we assumed that the coverage values of its constituent scaffolds is normally distributed, and thus eliminated HGTs on scaffolds that had coverage values greater than one standard deviation from the mean coverage of its respective host MAG. For prokaryotic and viral MAGs, if a putative HGT's scaffold had coverage values outside one standard deviation from the MAG's mean in > 2 samples (denoted in red in Table S1), they were discarded. If the HGT was outside the cutoff in 1 or 2 samples (denoted in yellow) then it was retained and scrutinised more intensely during the functional analysis steps (see below). If the HGT was inside the cutoff for all samples (denoted in green), then it was considered to have good coverage congruence with its host MAG.

### 2.4 Building Phylogenies for Protein‐Coding Results

A phylogeny was constructed for each putative HGT sequence that had a blastx hit to the nr database (i.e., is putatively protein‐coding (Buchfink, Reuter, and Drost 2021)). The nucleotide sequence of each putative coding HGT region was translated using Expasy “translate” tool to determine the best open reading frame for downstream analysis (Gasteiger et al. 2003). The best amino acid sequence per HGT region was queried against the NCBI nr database using diamond blastp v2.1.2.156 (Buchfink, Reuter, and Drost 2021). We then aligned the protein sequences of the resulting nr hits and the query ORF using MAFFT‐linsi v7.490 (Katoh and Standley 2013) and built single‐gene maximum likelihood (ML) trees using IQ‐TREE v1.6.12 (Nguyen et al. 2015), with automated model selection and node support estimated from 1000 ultrafast bootstrap replicates (Hoang et al. 2018).

## 3 Results

### 3.1 k‐mer Analysis and Filtering

Tf‐idf analysis returned 13,060 raw results. Step 1 of filtering (by size, < 100‐bp) reduced this set to 269 results. Following this step, we performed additional filtering to remove any overlap between the viral and cellular MAGs. This step was necessary for our dataset due to the different binning approaches used for the different domains (i.e., the cellular and viral MAGs were published in different studies), however, this step is not universally required. Additionally, we eliminated all virus‐to‐virus HGT results because we cannot exclude false positives due to homology in viral bins that lack precise taxonomic annotations. In total, this step eliminated 68 results, leaving 201 HGT candidates. Next, step 2, which was employed to remove scaffolds that were almost completely covered by putative HGT regions, and thus cannot be assessed and validated as belonging to the host MAG, reduced the list further to 186 sequences. Step 3 reduced the results nearly 60% by eliminating 101 sequences that had a majority composition of simple repeats, which, due to their ubiquity, cannot be easily validated as having arisen in a genome via HGT. For this step, scaffolds with > 50% simple repeat coverage were removed. We also eliminated 15 HGTs which covered regions composed predominantly of scaffolded sequences (i.e., regions with high “N” content), which resulted in 70 putative HGT events remaining.

The results of step 4 are useful, but not straightforward to interpret. In this step we queried the non‐HGT region of each HGT‐containing scaffold against the NCBI nr database using BLASTx. If the resulting top hit was to a protein from an organism from the same taxonomic class as the recipient MAG, it provided additional validation for this result, however, if the top hit was to the same class as the donor lineage, this indicates that it could be a false positive, or an artefact introduced during assembly or binning. Based on this procedure, 13 results were eliminated. Fifteen of the 70 results had no blast hits, and thus could not be assessed in this step. Furthermore, some of the results with hits were hard to interpret due to: (1) their association with genomes or MAGS in ‘candidatus’ organisms, (2) genomes that were not particularly closely related to either donor or recipient class, or (3) the relationship of our MAG with the organism associated with the hit is unclear because of under‐sampling or incomplete knowledge of prokaryote taxonomy. HGTs that fall under any of the ambiguous cases described above were retained to avoid over‐filtering the results. This step left 54 putative HGT candidates that were then assessed for GC content (Text S3).

In the final filtering step (step 5), we re‐mapped the raw DNA reads from each library (12 in total: 4 creek biofilm, 4 endolithic, 4 soil) against the MAGs used for tf‐idf analysis (see methods for how algal MAGs were analysed) and compared the average fold coverage per library per MAG for each HGT; see Table S4. From this step, 18 results had aberrant coverage values and were subsequently eliminated, leaving 39 results which passed all filtering steps.

### 3.2 Functional Analysis and Phylogenies

**FIGURE 2:**
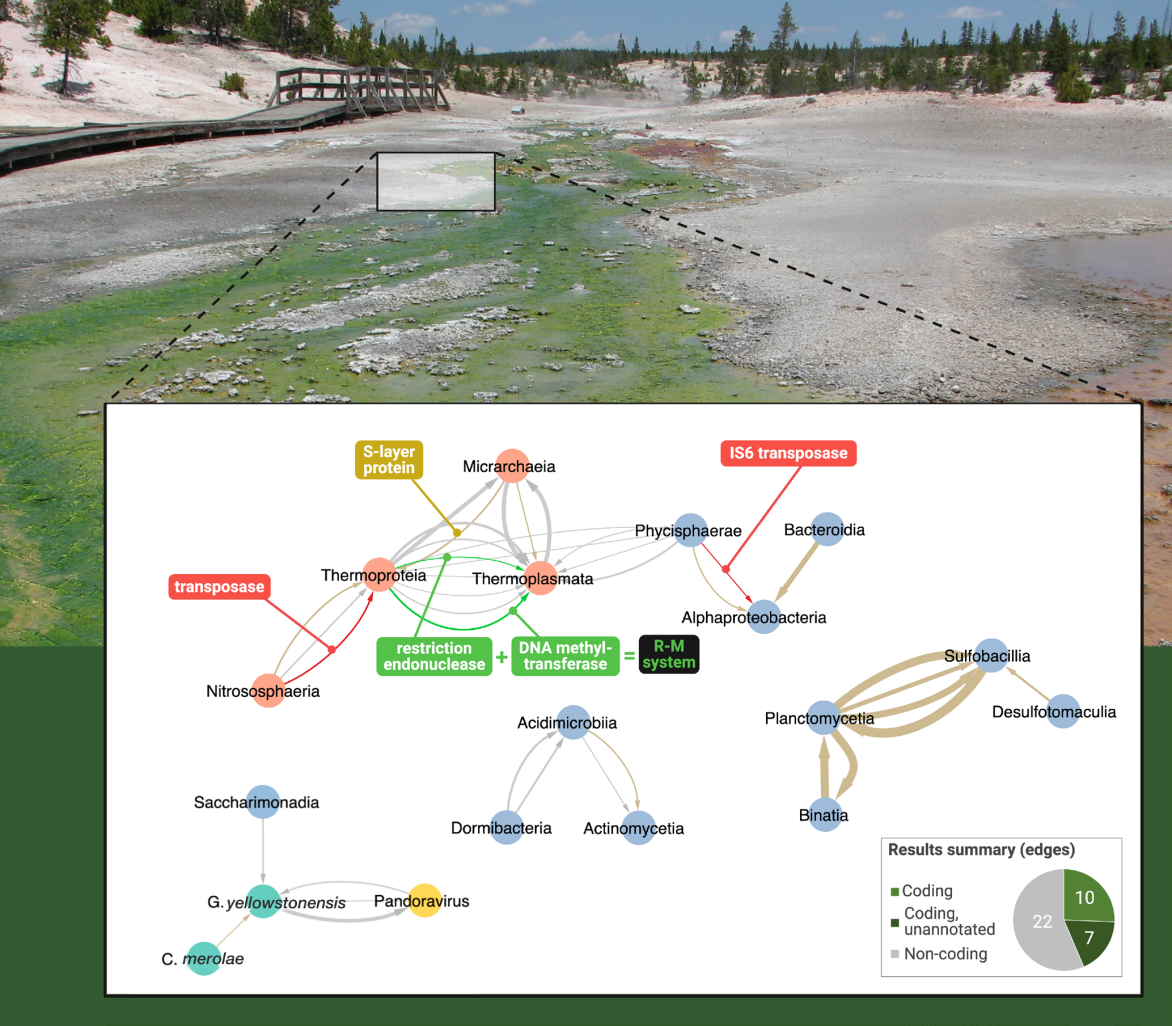
Network of the final putative HGTs identified in this study, created in Cytoscape v.3.10.1 (Shannon et al. 2003). Nodes represent MAGs grouped by taxonomic class (for prokaryotes and viruses). Bacterial classes are shown as blue nodes, archaeal classes as orange nodes, the viral MAG that passed validation as a yellow node, and each Cyanidiophyceae species as its own green node. Edges represent the filtered putative HGT events detected between each group, with the directionality of the transfer indicated by the direction of the arrow. Edges coloured red indicate sequences putatively encoding transposases. Edges in green are the restriction endonuclease and site‐specific DNA methyltransferase. Tan edges indicate other protein‐coding sequences. Edge thickness represents the length of the putative HGT, i.e., thinner edges means shorter HGT regions (minimum = 100‐bp) and thicker means longer HGT regions (maximum = 946‐bp). Photo of Yellowstone National Park from D. Bhattacharya, representing the biological context that exists for facilitating these genomic interactions. Made with Biorender.com.

**FIGURE 3:**
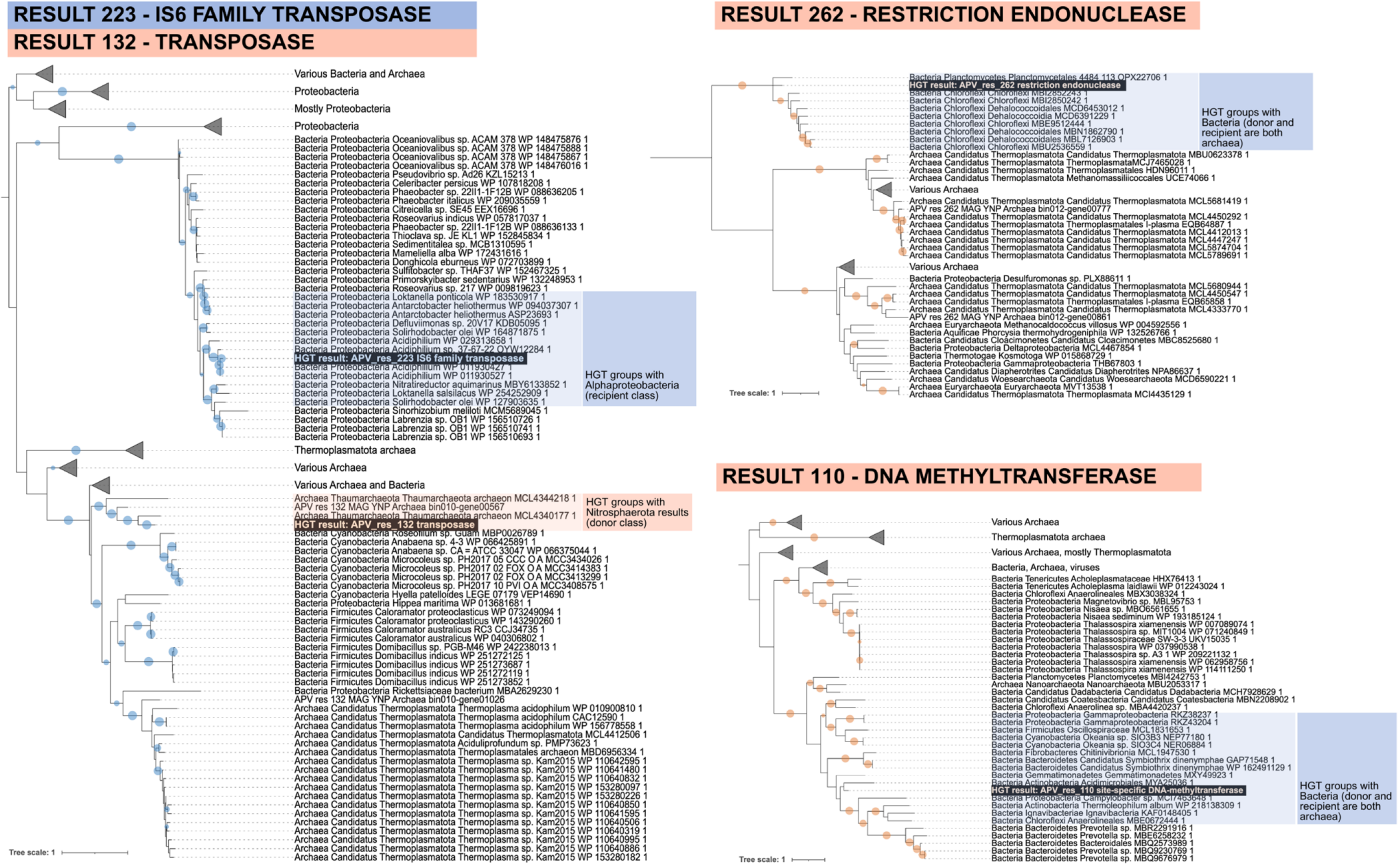
Three single‐gene amino acid phylogenies of major protein‐coding HGTs with annotated functions potentially related to DNA transfer (the other 13 phylogenies of protein‐coding HGTs can be found in Figures S1 and S2). On the left side of the figure is a single phylogeny including both the IS6 family transposase found within Bacteria (top) and the second, unrelated transposase found within Archaea (bottom). On the right side of the figure are phylogenies for a restriction endonuclease (top) and a site‐specific DNA methyltransferase (bottom), both transferred between the same two archaeal classes (Thermoproteia to Thermoplasmata). Circles on branches represent bootstrap values between 90 and 100. Phylogenies were formatted and edited in iTol v5 (Letunic and Bork 2021).

Of the 39 final HGT candidates used to build the network in Figure 2, 17 had shared homology with proteins in NCBI's nr database and were considered to be putatively protein‐coding (Table 1); the remaining 22 HGTs without hits were considered non‐coding, based on the available information. Of the 17 coding results, 10 have top hits to proteins with inferred functions and 7 have top hits to “hypothetical” proteins, and thus have unknown functions (see Figure 2, pie chart). All 17 protein‐coding HGTs were translated and used for phylogenetic analysis (Figures 3, S1, and S2), however, one of the unannotated proteins (APV_res_80) did not have a phylogeny constructed due to only one hit being returned from the nr database, providing an insufficient number of sequences for phylogenetic inference. Summary information on best hits and percent identity of these results can be found in Table 1.

**TABLE 1:** Final list of HGT‐derived protein‐coding regions and statistics about the encoded protein retrieved from the NCBI nr database. These results are 17 out of 39 total, therefore ca. one‐half of the final HGT candidates are putatively non‐coding sequences that can be found in Table S1.

Res ID	L	Donor	Recipient	NR annotation	PID	Acc. No.
35	946	Sulfobacillia	Planctomycetia	NAD‐dependent epimerase/dehydratase family protein from nitrospira bacteria	69.9	MBP0133249.1
40	826	Planctomycetia	Sulfobacillia	GH1|GH5_19 from thermomicrobiales bacteria	78.4	CAA9574979.1
41	812	Planctomycetia	*Binatia*	Hypothetical protein from deltaproteobacteria	63.8	MBV8137480.1
45	685	Planctomycetia	Sulfobacillia	GH1 family beta‐glucosidase from *Thermogemmatispora carboxidivorans*	61.1	WP081839020.1
51	519	Bacteroidea	Alphaproteo	Hypothetical protein from *Candidatus Amoebophilus* sp. 36–38	67.3	OJW69577.1
54	452	Planctomycetia	Sulfobacillia	ABC transporter substrate‐binding protein from Bacillota bacterium	69.1	MCL4520359.1
80	230	Desulfotomaculia	Sulfobacillia	Hypothetical protein from *Rhizobium etli* CNPAF512	74.2	EGE57262.1
110	190	Thermoproteia	Thermoplasmata	Site‐specific DNA‐methyltransferase from acidimicrobiales bacterium	72.6	MYA25036.1
118	178	*Nitrososphaeria*	Thermoproteia	Hypothetical protein from *Nitrososphaerota archaeon*	78.0	NHV98182.1
119	178	Micrarchaeia	Thermoproteia	S‐layer protein from *Candidatus* Marsarchaeota archaeon	67.8	MCL5099690.1
132	167	Nitrososphaeria	Thermoproteia	Transposase from Nitrososphaerota archaeon	62.1	MCL4340177.1
155	150	Acidimicrobiia	Actinomycetia	DNA‐directed RNA polymerase subunit beta from *Mycobacterium* sp.	100	NDA50076.1
164	144	Phycisphaerae	Alphaproteo	Hypothetical protein from *Acidiphilium* sp. 34–60‐192	90.5	OZB37901.1
182	132	Micrarchaeia	Thermoplasmata	YddF family protein from *Thermoplasma* sp. Kam2015	52.4	WP110641154.1
223	107	Phycisphaerae	Alphaproteo	IS6 family transposase from *Acidiphilium cryptum* JF‐5	97.1	WP011930527.1
APV_241	103	*C. merolae*	*G. yellowstonensis*	Hypothetical protein from *Colletotrichum graminicola* M1.001	90.9	EFQ36859.1
APV_262	102	Thermoproteia	Thermoplasmata	Restriction endonuclease from Dehalococcoidales bacterium	72.7	MCD6453012.1

Abbreviations: Acc. No., “NCBI accession number”; L, length; PID, “percent identity”; ResID, “result ID”.

Among the 10 protein‐coding HGTs that had functional annotations assigned, two were transposases (although the phylogeny in Figure 3 supports that they are different transposases) and one was a restriction endonuclease (Figure 3). There was also a site‐specific DNA methyltransferase (Figure 3), an NAD‐dependent epimerase/dehydratase family protein, an ABC transporter substrate‐binding protein, a GH1 family betaglucosidase, another result annotated as “GH1 | GH5_19 protein”, a DNA‐directed RNA polymerase subunit beta, and an archaeal S‐layer protein. Each of these results are shown in Figure S1 unless indicated otherwise. To provide support for these phylogenies, any other proteins with similar annotations within the MAGs involved in each of these transfers (e.g., other transposases, other methyltransferases, dehydratases) were included in the alignment and tree‐building process to check if the result groups with these similar proteins from the same assembly (i.e., are native proteins and probable false positives) or with other organisms (supporting their origin in the assembly via HGT). In most cases, the putative HGT result grouped with sequences outside of the MAG class from which it came, adding more weight to their origin via HGT. One exception was APV_res_132 (a transposase; Figure 3), although the MAG sequence that it was grouped near is ‘gene 00567’, and the HGT result is part of ‘gene 00568’, indicating that this HGT is likely duplicated, rather than a false positive. Also, due to our choice to collapse nodes not containing the result of interest to create small figures for this manuscript, most of these MAG sequences are not visible in Figure 3, however, they are in the Newick tree files provided in Appendix S1 and can be viewed in their entirety in any tree visualisation tool. Algal‐specific results and discussion can be found in Text S4.

### 3.3 Microbial Community as a Network

The final HGT results were used as input for Cytoscape (Shannon et al. 2003) to build the network shown in Figure 2. Here, the individual connections between genomes described above can be viewed within a community context. All MAGs in this study represent organisms found within centimetres of each other in Lemonade Creek and some are part of a physically connected biofilm, making the direct and indirect interactions that drive DNA transfer inevitable. HGT is a process that connects two organisms to each other, however most studies only focus on the transfer itself and not the ecological context under which the transfer occurred. By performing TF‐IDF analysis and visualising the results as a network, we can visually identify trends in the data and connect them to ecology. The network also highlights that most transfers occur within a domain of life. That is, there are more Archaea to Archaea (15) and Bacteria to Bacteria (14) transfers than Bacteria to Archaea (5), Archaea to Bacteria (0), Bacteria to eukaryote (1), and eukaryote to/from viruses (3).

## 4 Discussion

### 4.1 Recent DNA Transfers Are Mostly Gene‐Agnostic, or Linked to Transfer Mechanisms

Of the final HGT results, 22 of 39 (56%) were putatively non‐coding DNA. This is significant, suggesting that HGT is not necessarily a singularly functional or adaptive process. In fact, these results (albeit preliminary) suggest that the majority of transferred DNA (detected after a conservative filtering protocol) may represent non‐coding, potentially non‐functional regions, or DNA which encodes regulatory factors or unknown functional elements (Oren et al. 2014). Traditional phylogeny‐dependent analyses that uncover the relics of adaptive HGTs are the easiest to find and rationalise but they do not tell the whole story. Genetic transfer is likely a common and continuous process in all domains of life (Huang 2013), with extensive prokaryotic pangenomes described (Lukjancenko, Wassenaar, and Ussery 2010; Cummins et al. 2022) and cases of eukaryotic gene transfers compensating for gene loss during primary endosymbiosis and parasitism that indicate that HGT is so pervasive that it becomes compensatory in cases of gene loss and can save lineages (Van Etten and Bhattacharya 2020; Nowack et al. 2016; Yang et al. 2016). But there is much to learn from the quantity and quality of putatively non‐coding DNA that, based on the results of this study, is transferred in significant proportions, similar to that of putatively coding DNA. Additional investigation of non‐coding HGTs can be used to quantify background levels of genetic transfer across many communities, that is, levels of recent DNA transfers that can be captured by the TF‐IDF method that may not be fixed over long timescales. This will shed light on the extent of genetic connectivity between ecologically linked genomes and provide context to adaptive, protein‐coding transfers and their rarity and evolutionary significance. That being said, identifying absolute rates and truly exhaustive HGT events remains a challenge, even with the advent of methods like TF‐IDF that identify more cases than traditional approaches that only investigate coding regions. DNA transfers between closely related organisms, ancient/ameliorated transfers, and transfers of particularly promiscuous sequences that may not have a clearly detectable foreign origin remain difficult to identify and thus, absolute rates of HGT are likely to be underestimated (Lloyd et al. 2018; Lynch and Neufeld 2015; Kloub et al. 2021).

If genomes *are* extensively connected as we hypothesize above, perhaps populations within a community serve as reservoirs of adaptive DNA sequences or those encoding mechanisms to receive and integrate foreign DNA for other organisms, like a community pangenome of shared resources. In this study, 17 of the 39 (44%) final candidates share homology with protein‐coding genes, although we did not yet explore if these sequences are complete or expressed in the genomes they were found. Interestingly, two protein‐coding transfers were transposases, and one was a restriction endonuclease. The first (APV_res_132) is a transfer within Archaea, from the Nistrosphaeria class to the Thermoproteia class. This sequence had high blastx percent identity (PID; up to 62.5%) to other transposases across Nitrosphaeria and various ‘candidatus’ class organisms in other Archaea (see Table 1 and Figure 3). The second transposase (APV_res_223) was transferred within Bacteria, from Alphaproteobacteria to Phycisphaerae, and hits to other sequences (PID up to 97.1%) annotated as IS6 family transposases (see Table 1 and Figure 3). The validity of this result is less clear based on the phylogeny because the transfer is situated near other alphaproteobacterial sequences in the tree. The restriction endonuclease (APV_res_262) was transferred within Archaea, from the Thermoproteia to Thermoplasmata. Transposases are enzymes that move mobile DNA segments (transposons) from one part of a genome to another or even from genome to genome (Calos and Miller 1980). Both transposases and endonucleases have been strongly implicated in HGT, with specific cases of microbial resistance genes jumping between genomes in prokaryotes (Luo et al. 2012; Cuecas, Kanoksilapatham, and Gonzalez 2017; Haudiquet et al. 2022). There is another result (APV_res_110) also transferred from Thermoproteia to Thermoplasmata that is a putative site‐specific methyltransferase. Interestingly, both restriction endonucleases and methyltransferases are part of the restriction‐modification system that moderates HGT (Oliveira, Touchon, and Rocha 2014; Tokuda and Shintani 2024). This system can act like an innate immune system for prokaryotes that aids in degrading non‐self DNA (Oliveira, Touchon, and Rocha 2016), a useful function in an organism that accepts many HGTs that may be beneficial or deleterious, which appears to be more common among Archaea in this dataset (Figure 2), and particularly in the Thermoplasmata that is the recipient of these two transfers and is the recipient of the most validated HGTs compared to all other classes in this analysis. Interestingly, there seems to be a directional flow of these mobile DNA (transposase and endonuclease) functions across the archaeal sub‐network (Figure 2) that may potentially suggest a HGT mechanism requiring future investigation.

Most of the final HGTs identified in our analysis were between different classes of Archaea. Interestingly, Cong et al. used clique analysis to look for genetic exchange communities within a prokaryotic dataset, composed of completely sequenced prokaryotic genomes not linked by shared environment (Cong et al. 2017). They found much less HGT among Archaea than among Bacteria. They suggest that this may either be because Archaea exchange less DNA than Bacteria or because many Archaea live in highly specialised environments (Cong et al. 2017). Our study, which is focused on a specialised extremophilic environment, shows that Archaea, more than any other group within the analysis, had the highest incidence of validated HGT, despite having far fewer MAGs than Bacteria to examine. This emphasises why studying HGTs between ecologically unlinked genomes may be underestimating or biasing our understanding of this process in nature.

Other protein‐coding transfers with annotations include a NAD‐dependent epimerase/dehydratase family protein, transferred from Sulfobacillia to Planctomycetia (APV_res_35). This protein in *P. carotovovorum* and its homologue in *E. coli* have been shown to be involved in the biosynthesis of exopolysaccharides and enhances biofilm formation (Islam et al. 2019). Similarly, another transfer was an archaeal S‐layer protein, between Micrarchaeia and Thermoproteia (APV_res_119). S‐layer proteins can make up the outer layer of the cell in Archaea and contribute to surface attachment, although their role in biofilm formation is still under investigation (Li Wong et al. 2023). There was also an ABC transporter substrate‐binding protein transferred from Planctomycetia to Sulfobacillia (APV_res_54) which is likely to be involved in membrane transport (Maqbool et al. 2015). There is a DNA‐dependent RNA polymerase subunit beta homologue that was identified as transferred from Acidimicrobiia to Actinomycetia (APV_res_155), that may possibly be implicated in DNA modification (Iyer, Koonin, and Aravind 2004). Last, two proteins were identified, both from Planctomycetia to Sulfobacillia (APV_res_45 and APV_res_40) annotations to GH1 family beta‐glucosidase and “GH1 | GH5_19”, respectively. Beta‐glucosidase proteins generally function to catalyse the hydrolysis of cellobiose and oligosaccharides to produce glucose (Singh, Verma, and Kumar 2016). Based on accession numbers of the proteins nearest the APV_res_40, (GH1 | GH5_19) sequence in the tree (Figure S1), it seems that this protein is most closely related to beta‐galactosidase, which is part of the bacterial lac operon and produces galactose and glucose for glycolysis (Juers, Matthews, and Huber 2012). HGT is easiest to understand through the lens of adaptive, protein‐coding genes. It is not hard to find advantages to having any of the aforementioned genes, particularly in a geothermal environment where genomes are under selection for reduced size, but must still deal with emerging environmental stresses. However, it is likely that the organisms currently residing here are generally well‐adapted to these conditions and in times of environmental stability, there is a weak drive for HGT fixation. Additional discussion of algae and virus results can be found in Text S5.

### 4.2 Study Limitations

The tf‐idf program outperforms other alignment‐free HGT‐mining programs such as ALFY (Domazet‐Lošo and Haubold 2011a, 2011b) in both real and simulated datasets (Cong, Chan, and Ragan 2016; Bernard et al. 2019; Cong et al. 2017). However, this assessment was performed on complete prokaryote genomes generated from pure cultures, not environmental metagenomics data from eukaryote‐dominated habitats. Additionally, this program identifies DNA HGT events, and does not consider protein sequences, as do traditional HGT identification approaches. Thus, the results from tf‐idf must be considered differently, and care must be chosen when deciding on the best method for a particular study.

For example and as a control, the Cyanidiophyceae red algae have many well‐documented cases of HGT that have been identified, mainly via orthogroup analysis (Rossoni et al. 2019; Schönknecht, Weber, and Lercher 2014; Van Etten et al. 2023; Qiu et al. 2013; Schönknecht et al. 2013), however, none of those known (protein‐coding) HGTs were identified in the two Cyanidiophyceae MAGs ( *G. yellowstonensis* and *C. merolae*) used in this study by tf‐idf. There are two main reasons potentially underlying this result. The first is that the donor organisms may not currently cohabit this environment or may not be present in the assembled MAG data. As with all tools for HGT identification, tf‐idf can only identify HGTs between groups that are well represented and accurately presented in its input dataset. If a more taxa rich, complete dataset was to be used with tf‐idf, then it is expected that more results will be generated, presumably especially within virus data. Second, the known HGTs in Cyanidophyceae are ancient, having been acquired by the ancestors of these algae from donor taxa hundreds of millions of years ago, likely during periods of acute stress that created selective pressures favouring rapid adaptation (Rossoni et al. 2019; Van Etten et al. 2024; Yoon et al. 2017). Over time, the amelioration process, which leads to properties of the foreign DNA conforming to those of the recipient genome, will result in the accumulation of nucleotide differences between the HGT and the donor sequence. These differences will alter the *k*‐mer composition of the HGT region, preventing its identification at a DNA level using *k*‐mer‐based analysis (Lawrence and Ochman 1997; Callens, Scornavacca, and Bedhomme 2021). Amelioration has less of an effect on protein sequences due to stronger selective constraints on their sequence. Thus, if the identification of ancient HGT events is the goal of a study, then it is expected that only coding transfers can be identified, and a traditional phylogeny‐based method is preferable. Because of these considerations, we did not expect to find the known (ancient) Cyanidophyceae HGTs, but rather more recent HGT that may be specific to a particular lineage or environment.

This analysis was designed as a proof‐of‐concept for the application of tf‐idf to environmental metagenomic data which is often suboptimal. We believe that the number, quality, and types of the filtered HGT results produced by tf‐idf (discussed above; see also Figures 2, 3 and S1) prove its capacity to accurately identify DNA transfers in this type of data. Whereas short read‐based metagenomic data (used here) is highly informative of the content and abundance of microbes in an environment, it is unable to reliably produce complete or near complete microbial genomes which are required for optimal in‐depth HGT exploration (Gehrig et al. 2022; Kraft and Kurth 2020; Zhao et al. 2023). The increased prevalence of long‐read‐based metagenomics projects opens the door for follow‐up analysis using more complete and robust MAGs from a variety of different environments. We thus recognise that our study is somewhat limited by the use of short‐read data and consequently introduced the conservative validation pipeline (Figure 1) to partially remedy this shortcoming. Accordingly, and as is done with most HGT studies regardless of method, we present all results as “putative” HGTs which, if coding, have not yet been validated functionally, or otherwise, must be considered with a degree of caution due to the limitations highlighted here.

### 4.3 ‘Genome Connectivity’ as a Feature of Community Ecology

DNA transfer does not exist within a vacuum. Studying adaptive single protein‐coding transfers can be highly informative about the evolution of an organism, including the major selective forces that have driven its genome evolution; however, organisms do not respond to stress alone. Environmental adaptations exist across many levels of biological organisation, from individual genomes to entire ecosystems. Studying HGT can inform us of instances in time when two genomes were connected, with the existence of foreign DNA in a new host genome evidence of this ‘genome connectivity’. Because genomes of closely related organisms, as shown within this study (majority within‐domain transfers), and genomes of organisms in close associations (shown through the highly connected network resulting from this study of a single, biofilm‐based community; Figure 2) are known and shown to have higher incidence of transfer (Bolotin and Hershberg 2017; Abe, Nomura, and Suzuki 2020), we propose that HGT‐driven relationships can be used in future studies to predict ecological relationships. This connects perhaps the lowest level of biological organisation (the genome within an individual cell within a population) to one of the highest levels: the ecosystem. We cannot understand the extent of genome connectivity from coding transfers alone. Techniques such as tf‐idf allow us to uncover exhaustive (coding and non‐coding) DNA transfers, providing a means to observe this process in action in the environment, and understand how HGT reflects or mediates organismal interactions that affect ecosystem structure. Furthermore, it is well‐known that communities under selection for genome streamlining (such as the YNP geothermal springs) experience increased cooperation or co‐dependence between divergent lineages. This observation has led to hypotheses such as the Black Queen Hypothesis (Fullmer, Soucy, and Gogarten 2015; Souza, Irie, and Eda 2022) and the Integrated HGT Model of eukaryote genome evolution (Stephens et al. 2024; Van Etten et al. 2024) which attempt to explain how different organisms can participate in discrete steps of a larger metabolic process (e.g., detoxification pathways) to compensate for the lack of a single organism possessing the complete pathway. These hypotheses are central to HGT because DNA transfers offer a rapid mechanism for organisms to become integrated into local community ecology through gene sharing. Tf‐idf provides a practical approach to identify such biotic interactions from the genome‐up, rather than from ecology down. Future studies that use long‐read metagenomic or single‐cell sequencing techniques to look at diverse environments will make it possible to identify global trends in HGT and to elucidate how genome connectivity begets ecology. In conclusion, the ability to identify recent DNA transfers not only connects community members to each other but also provides insights into the mechanisms underlying this process, which are obscured by the passage of time and are otherwise undetectable when only considering ancient adaptive protein‐coding HGTs.

## Author Contributions

**Julia Van Etten:** conceptualization, data curation, formal analysis, investigation, methodology, funding acquisition, visualization, project administration, writing – review and editing, writing – original draft. **Timothy G. Stephens:** data curation, formal analysis, software, methodology, writing – original draft, writing – review and editing. **Debashish Bhattacharya:** conceptualization, investigation, funding acquisition, project administration, writing – review and editing, supervision.

## Conflicts of Interest

The authors declare no conflicts of interest.

## Supporting information


**Data S1.** Supporting Information.

## Data Availability

The non‐redundant prokaryotes, other eukaryotes, and viral MAGs used for this analysis are available from https://zenodo.org/doi/10.5281/zenodo.12710562. The Cyanidiophyceae MAGs generated for HGT analysis are available from https://zenodo.org/records/13651146. The modified version of the tf‐idf program used in this research is available from https://github.com/TimothyStephens/TF‐IDF.
