# Supplementary material for: Genetic Transfer in Action: Uncovering DNA Flow in an Extremophilic Microbial Community
Source: Environ Microbiol. 2025 Feb 3;27(2):e70048. doi: 10.1111/1462-2920.70048 (PMC11790422; doi:10.1111/1462-2920.70048)
Supplement: Supplementary file 1 — Data S1. Supporting Information. [file EMI-27-e70048-s001.docx]

**APPENDIX**

**Supplementary text, figure, tables, and file locations for: Genetic transfer in action: uncovering DNA flow in an extremophilic microbial community**

Julia Van Etten^1*^, Timothy G. Stephens^1^, and Debashish Bhattacharya^1^

^1^Department of Biochemistry and Microbiology, Rutgers, The State University of New Jersey, New Brunswick, NJ 08901, United States of America

*^*^Correspondence:* Julia Van Etten, [julia.vanetten@rutgers.edu](mailto:julia.vanetten@rutgers.edu)

**Supplementary text 1:**

*Metagenomic data*

The metagenome assembled genomes (MAGs) used for tf-idf analysis were generated by Stephens et al. (2024) [a] and Benites et al. (2023) [b]. A comprehensive description of sample collection, extraction, processing, and MAG construction is provided in each publication. Briefly, three Lemonade Creek, YNP environments (creek biofilm, endolithic, and soil) were sampled (n=4) for metagenome sequencing. The metagenome reads from each sample were independently cleaned and assembled following the JGI’s inhouse pipeline (SOPs 1064 and 1077). Three sets of MAGs were produced from the assembled samples: prokaryotic, Cyanidiophyceae (extremophilic, unicellular red algae that dominate these habitats), and viral.

Prokaryotic MAGs

Prokaryotic MAGs were constructed independently for each of the 12 metagenome samples before being combined into a single set of non-redundant (98% Average Nucleotide Identity [ANI]; approximately at the strain level) MAGs that were taxonomically classified and used for downstream analysis. At each stage of the binning process, care was taken to remove putative contaminant (e.g., Cyanidiophyceae) scaffolds from the prokaryotic MAGs, and to clean or remove any scaffolds arising from chimeric bins. This produced a final set of 174 non-redundant prokaryotic MAGs (138 Bacteria, 36 Archaea; Table S3). See Stephens et al. (2024) [a] for an exhaustive description of all tools and parameters used for this analysis.

Cyanidiophyceae MAGs

The assembled metagenome scaffolds for each sample were compared against a database consisting of 16 Cyanidiophyceae reference genomes (Table S2) using BLASTn v2.10.1 (‘-max_target_seqs 2000 -evalue E ≤ 10-10 -dust no -soft_masking false’); repeat masking in BLAST was disabled to allow for better alignment of repetitive regions. Only hits which had >90% identity were retained for downstream analysis and hits which overlapped along each scaffold were merged into a single feature. The coverage of each scaffold by the resulting merged BLASTn hit features was calculated using bedtools v2.29.2 [c]. Scaffolds which had >10% of their bases covered by merged BLASTn hits were classified as putative Cyanidiophyceae sequences; 10% coverage was chosen to prevent over-filtering the data and removal of genuine instances of HGT from the results. Additionally, all downstream putative HGTs underwent extensive validation, reducing the chance that any false positive HGTs making it into the final results. Sequences recruited into the prokaryotic MAGs were removed from the Cyanidiophyceae MAGs before downstream analysis. The putative origin of the Cyanidiophyceae scaffolds were assigned using the top hit (i.e., the hit with the highest bitscore) for each scaffold. That is, a scaffold was assumed to have originated from the same (or a close relative) species as its top hit. The majority of scaffolds had top hits to the *Cyanidioschyzon merolae* 10D and *Galdieria yellowstonensis* YNP5587.1 reference genomes — isolates similar to these two species that are known to inhabit these sites [a]. The metagenomic sequences attributed to each species in each sample are hereinafter referred to as the *C. merolae*10D-like and *G. yellowstonensis* YNP5587.1-like MAGs. The assembly quality and completeness of the extracted Cyanidiophyceae metagenome scaffolds was assessed using BUSCO v5.0.0 [d] (‘--mode genome’) using the ‘eukaryota_odb10’ dataset. Given the significant number of sequences identified in the algal MAGs from each sample, a single representative MAG (the one with the highest BUSCO completeness) per environment for each of the algal strains (*C. merolae* 10D-like and *G. yellowstonensis* YNP5587.1-like) was used for tf-idf analysis. This strategy (one algal MAG per environment) was designed to account for environment specific DNA transfers and to provide multiple genomes for tf-idf analysis, because it is designed to work with a cohort of genomes in each group. The algal MAGs used for downstream analysis were from the Soil4, CreekBiofilm4, and Endolithic4 (S4CB4E4) samples. Henceforth, the group comprising the S4CB4E4 *G. yellowstonensis* YNP5587.1-like MAGs is referred to as the “*Galdieria*” group, and the S4CB4E4 *C. merolae* 10D-like MAGs is referred to as the “*Cyanidioschyzon*” group. This approach does not significantly impact the HGT results returned by the program but influences validation of the results which require more sophisticated validation steps (see below).

Viral MAGs

Viral operational taxonomic units (vOTUs; i.e., putative viral scaffolds) were identified in each of the 12 metagenome samples using a variety of virus classification tools and databases, allowing for the broadest range of taxonomic classifications. The vOTUs from each scaffold were merged into a non-redundant set (> 95% average nucleotide identity and > 85% alignment fraction), which were then binned into putative viral MAGs and assigned taxonomic classifications. This produced a final set of 25 non-redundant viral MAGs which had non-ambiguous taxonomic classifications, i.e., they had taxonomic classifications that were not listed as “unassigned”. See Benites et al. (2024) [b] for an exhaustive description of the tools used for this analysis.

1. Stephens, T.G., Van Etten, J., McDermott, T., Christian, W., Chaverra, M., Gurney, J., Lee, Y., Kim, H., Cho, C.H., Chovancek, E. and Westhoff, P., 2024. Community-wide interactions sustain life in geothermal spring habitats. *bioRxiv*, pp.2024-09.
2. Felipe Benites, L., Stephens, T.G., Van Etten, J., James, T., Christian, W.C., Barry, K., Grigoriev, I.V., McDermott, T.R. and Bhattacharya, D., 2024. Hot springs viruses at Yellowstone National Park have ancient origins and are adapted to thermophilic hosts. *Communications Biology*, *7*(1), p.312.
3. Quinlan, A.R. and Hall, I.M., 2010. BEDTools: a flexible suite of utilities for comparing genomic features. *Bioinformatics*, *26*(6), pp.841-842.
4. Manni, M., Berkeley, M.R., Seppey, M., Simão, F.A. and Zdobnov, E.M., 2021. BUSCO update: novel and streamlined workflows along with broader and deeper phylogenetic coverage for scoring of eukaryotic, prokaryotic, and viral genomes. *Molecular biology and evolution*, *38*(10), pp.4647-4654.

**Supplementary text 2:**

Additional filtering: Following step 1, putative HGT results from viral MAGs were further validated by comparing (using BLASTn) each HGT-containing scaffold against the prokaryotic and algal MAGs. This step was performed because the prokaryotic and algal MAGs were produced in a separate study to the viral MAGs, thus an additional filtering step was implemented to remove any (rare) scaffolds classified as both cellular and viral by the two workflows. We also chose to eliminate all virus-to-virus results because it is challenging to confidently classify the vOTUs containing putative HGTs using a BLASTX search due to the viral taxonomy containing many unknowns. Thus, we cannot further validate these results under the conservative approach we have taken.

**Supplementary text 3:**

We used the stats.sh script from the BBMap package [30] to determine the GC-content of the scaffold containing the putative HGT versus the recipient MAG. This step was not to eliminate any candidates but as a qualitative measure to inform about the possible age and degree of amelioration of HGT candidates that pass validation. If foreign DNA enters a new genome (this trend is stronger in organisms that are not closely related), its GC-content likely initially differs from the recipient. However, as time goes on and the amelioration process occurs [31], properties of this DNA segment, including GC-content will become less distinguishable from the rest of the new genome. It is difficult to use this criterion as a basis for elimination because the method used in this study aims to identify sequences that may be very recent transfers, or transferred from genomes with similar GC content, so we expect a range of GC-content differences across the results.

The GC content step was informative, rather than reductive. Here, we compared the GC content of the scaffold containing the putative HGT to the GC content of the MAG in its entirety. If a DNA transfer is very recent and from another domain of life, the GC content is likely to differ significantly, however after amelioration, i.e., the process by which foreign DNA is integrated into a new genome, it will take on features of the recipient and become less conspicuous [e, f]. This means that different or similar %GC may not aid HGT validation, however, these data may provide insights into the timeline of the putative transfer. Of the final HGT candidate list (the outcome of step 5), only 4 out of 39 results had a %GC that fell outside of one standard deviation of the MAG mean, potentially indicating a relatively recent transfer event. These were results APV_res_51, APV_res_58, APV_res_80, and APV_res_224 which are transfers of Bacteroidea 🡪 Alphaproteobacteria, Thermoproteia 🡪 Micrarchaeia, Desulfotomaculia 🡪 Sulfobacillia, and Thermoproteia 🡪 Phycisphaerae, respectively. Alternatively, the 35 results with one standard deviation of the MAG mean could have arisen from donors with similar %GC to the recipient. This complexity is why we chose to not use %GC as a filtering criteria in this analysis.

1. Lawrence, J.G. and Ochman, H., 1997. Amelioration of bacterial genomes: rates of change and exchange. *Journal of molecular evolution*, *44*, pp.383-397.
2. Callens, M., Scornavacca, C. and Bedhomme, S., 2021. Evolutionary responses to codon usage of horizontally transferred genes in Pseudomonas aeruginosa: gene retention, amelioration and compensatory evolution. *Microbial genomics*, *7*(6).

**Supplementary text 4:**

*Algal results*

The Cyanidiophyceae red algae that dominate the biomass in YNP geothermal habitats are well-known for possessing a myriad of prokaryote-derived protein-coding HGTs of ancient origin [g]. Throughout this analysis, we did not find evidence of extensive HGT to the algal MAGs generated from these sites (*G. yellowstonensis* YNP5587.1-like and *C. merolae* 10D-like). Nonetheless, of the final 39 HGT candidates, one is a transfer from Saccharimonadia to *G. yellowstonensis*. Saccharimonadia are a newly discovered clade within the candidate phylum radiation (‘CPR infrakingdom’) composed of uncultivated bacteria with highly reduced genomes that are symbionts, obligate epibionts, or parasites [h]. An analysis like tf-idf is a useful approach to infer potential ecological associations worthy of future investigation. Perhaps Saccharimonadia-*Galdieria* symbiosis is one of these relationships. There were also three algal-virus results, two from the Pandoravirus vOTU's to *G. yellowstonensis* (APV_res_148 and APV_res_247) and one that is putatively in the other direction (APV_res_59). This could suggest a potential host-pathogen relationship between the algae and Pandoravirus. Pandoraviruses are widely documented to infect other protists [i], and in other studies, have been phylogenetically placed within Phycodnaviridae, a viral family known to infect algae [j]. Lastly and interestingly, there was a validated result (APV_res_241) transferred from the *C. merolae* MAG to the *G. yellowstonensis* MAG that has its highest homology with fungi, particularly *Colletotrichum graminicola*, and is placed between other fungi and protists (oomycetes and green algae) that may have also received this sequence via HGT (Fig. S2). It is unlikely that *C. merolae* actually donated this sequence to *G. yellowstonensis*, but because tf-idf only considers transfers between the genomes provided and there were no fungal MAGs included in this dataset, this result suggests a potential transfer to both algae in YNP (although *C. merolae* must have significantly more *k*-mers from this sequence than *G. yellowstonensis* for it to have been identified by our analysis) that implicates the fungal kingdom of life in the HGT record of the Cyanidiophyceae. Oomycetes are a group of Stramenopiles that have received many fungal DNA transfers [k], so this idea could be supported by their proximity in the phylogenetic tree to the fungal result (Fig. S2).

1. Rossoni, A.W., Price, D.C., Seger, M., Lyska, D., Lammers, P., Bhattacharya, D. and Weber, A.P., 2019. The genomes of polyextremophilic cyanidiales contain 1% horizontally transferred genes with diverse adaptive functions. *Elife*, *8*, p.e45017.
2. Albertsen, M., Hugenholtz, P., Skarshewski, A., Nielsen, K.L., Tyson, G.W. and Nielsen, P.H., 2013. Genome sequences of rare, uncultured bacteria obtained by differential coverage binning of multiple metagenomes. *Nature biotechnology*, *31*(6), pp.533-538.
3. Legendre, M., Fabre, E., Poirot, O., Jeudy, S., Lartigue, A., Alempic, J.M., Beucher, L., Philippe, N., Bertaux, L., Christo-Foroux, E. and Labadie, K., 2018. Diversity and evolution of the emerging Pandoraviridae family. *Nature Communications*, *9*(1), p.2285.
4. Yutin, N. and Koonin, E.V., 2013. Pandoraviruses are highly derived phycodnaviruses. *Biology direct*, *8*(1), pp.1-8.
5. Savory, F., Leonard, G. and Richards, T.A., 2015. The role of horizontal gene transfer in the evolution of the oomycetes. *PLoS pathogens*, *11*(5), p.e1004805.

**Supplementary text 5:**

*HGT in algae historically (modern vs. ancient)*

The results of this study primarily feature HGTs involving prokaryotes, which is to be expected because it is well-documented that Bacteria and Archaea exhibit a high frequency of genetic transfer, and these microbes make up most of the sequenced MAGs. However, two Cyanidiophyceae MAGs were also analyzed. These red algae make up over 80% of the biomass in certain sites within YNP [l] and studies have shown that approximately 1% of cyanidiophyceaen protein coding genes are of foreign origin [g, m, n]. Whereas the paucity of algal results from this study may seem counterintuitive, it actually supports the hypothesis that ancestors of the extant species and strains of these algae received many HGTs within a relatively short period of evolutionary time, following exposure to myriad of new stressors [o, p] that required rapid adaptation to survive. The lack of new DNA transfers into these genomes supports the idea that modern geothermal habitats like YNP are not acutely stressful to extent Cyanidiophyceae, however, our results show that there are still background levels of genetic transfer occurring even in the absence of strong selection for their retention. Furthermore, the algal results represent the only case of putative viral transfers that passed filtering, that is, three putative transfer events between *G. sulphuraria* and the Pandoravirus vOTU. This low incidence is likely an underestimation due to our conservative use of a small subset of viral genomic data from the YNP metagenome dataset as input for the tf-idf program.

1. Castenholz, R.W. and McDermott, T.R., 2010. *The Cyanidiales: ecology, biodiversity, and biogeography* (pp. 357-371). Springer Netherlands.
2. Hirooka, S., Itabashi, T., Ichinose, T.M., Onuma, R., Fujiwara, T., Yamashita, S., Jong, L.W., Tomita, R., Iwane, A.H. and Miyagishima, S.Y., 2022. Life cycle and functional genomics of the unicellular red alga Galdieria for elucidating algal and plant evolution and industrial use. *Proceedings of the National Academy of Sciences*, *119*(41), p.e2210665119.
3. Cho, C.H., Park, S.I., Huang, T.Y., Lee, Y., Ciniglia, C., Yadavalli, H.C., Yang, S.W., Bhattacharya, D. and Yoon, H.S., 2023. Genome-wide signatures of adaptation to extreme environments in red algae. *Nature communications*, *14*(1), p.10.
4. Van Etten, J., Stephens, T.G., Chille, E., Lipzen, A., Peterson, D., Barry, K., Grigoriev, I.V. and Bhattacharya, D., 2024. Diverse fates of ancient horizontal gene transfers in extremophilic red algae. *Environmental microbiology*, *26*(5), p.e16629.
5. Cho, C.H., Park, S.I., Huang, T.Y., Lee, Y., Ciniglia, C., Yadavalli, H.C., Yang, S.W., Bhattacharya, D. and Yoon, H.S., 2023. Genome-wide signatures of adaptation to extreme environments in red algae. *Nature communications*, *14*(1), p.10.

**Figure S1.** This figure is a composite of all single-gene amino acid phylogenies with a functional annotation, not included in Figure 3 of the main text. Below this figure are larger versions of all 9 gene trees (including those from Figure 3). Circles are bootstrap values between 90 and 100. All trees are midpoint rooted.


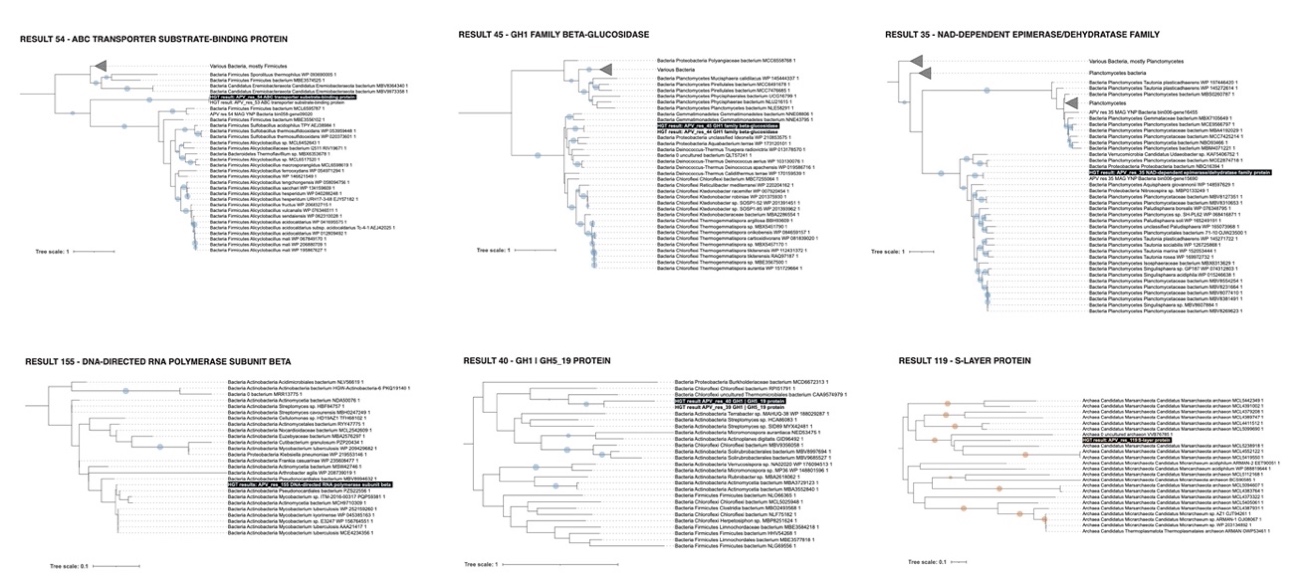


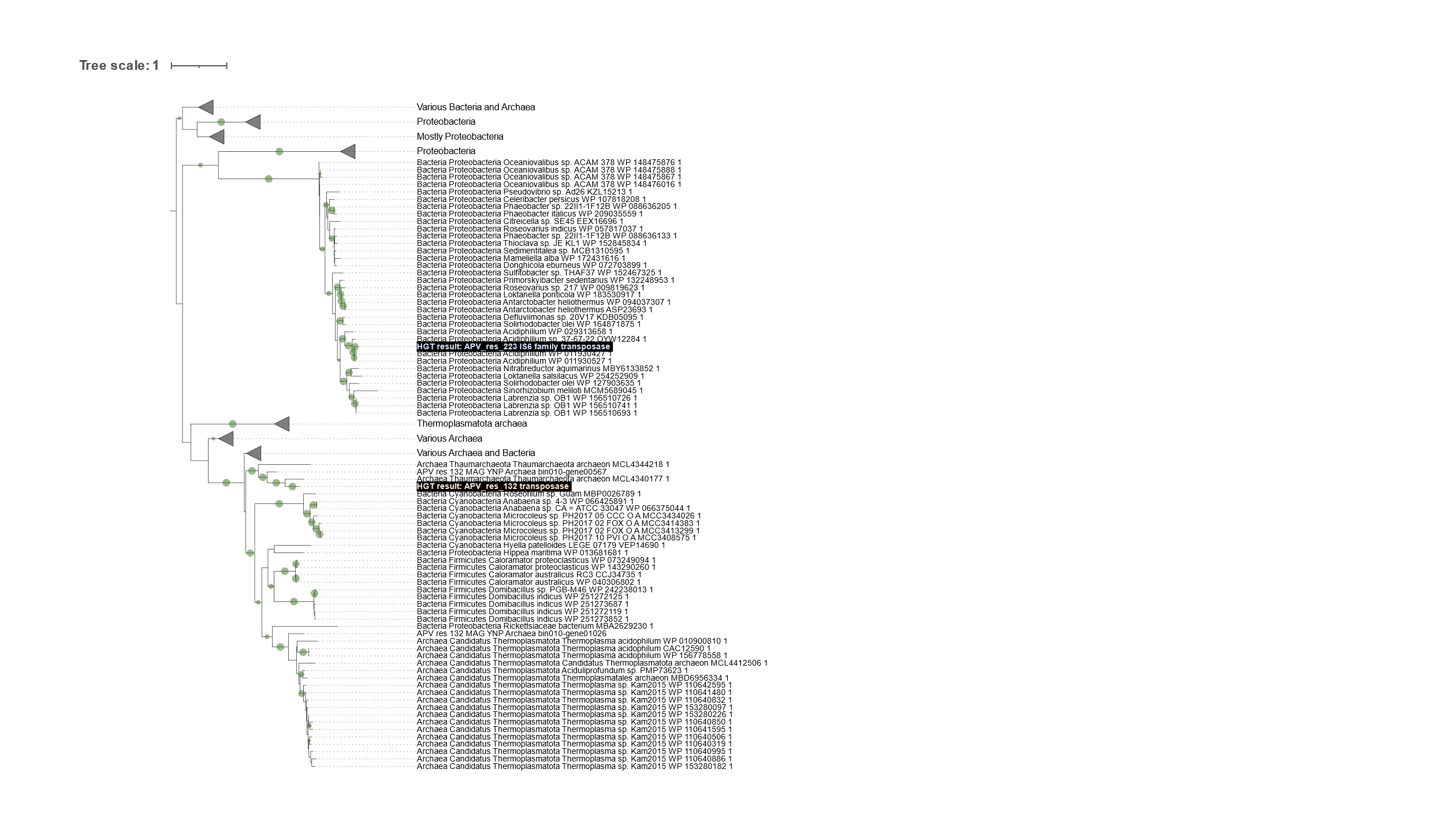


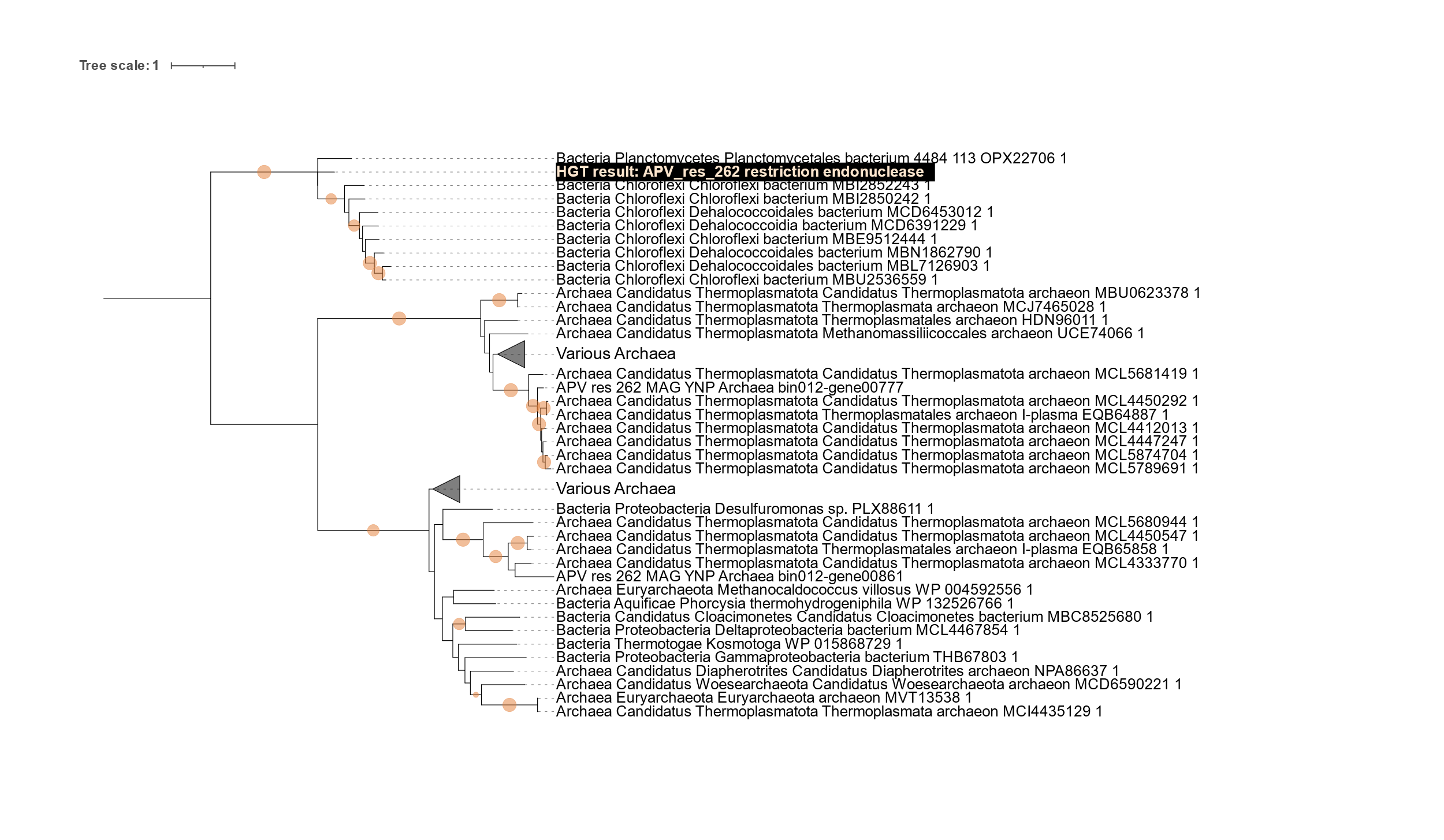


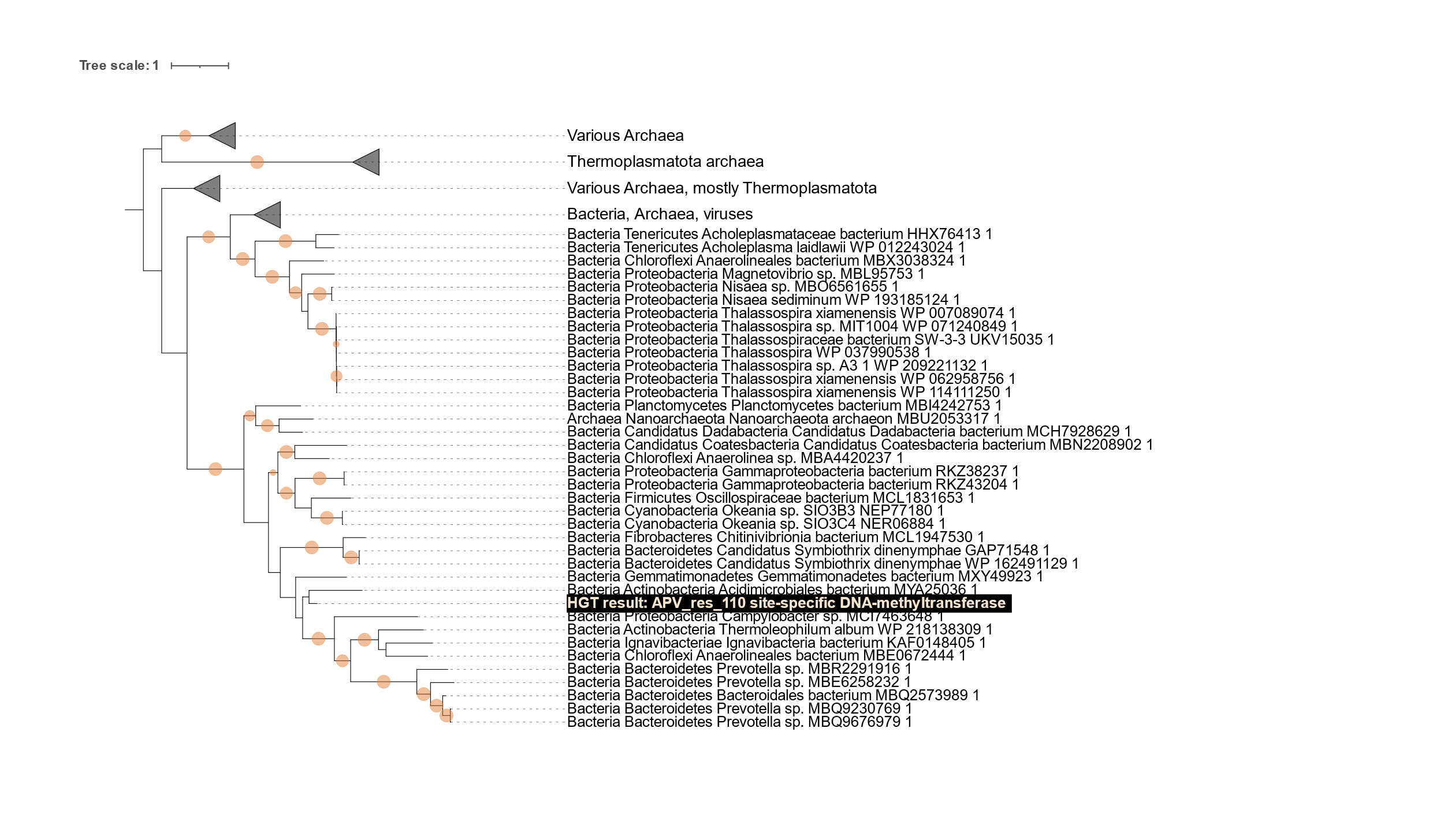


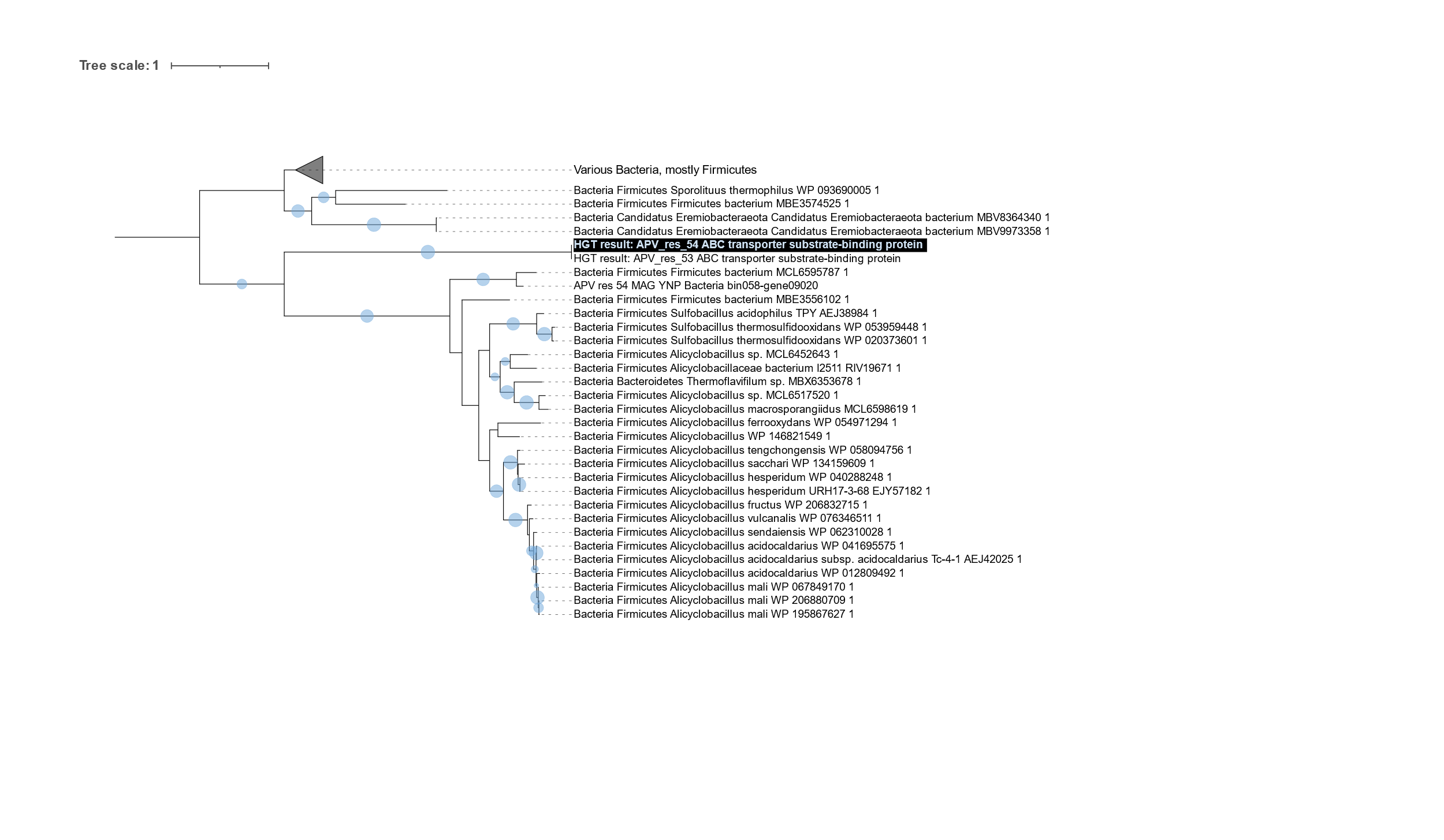


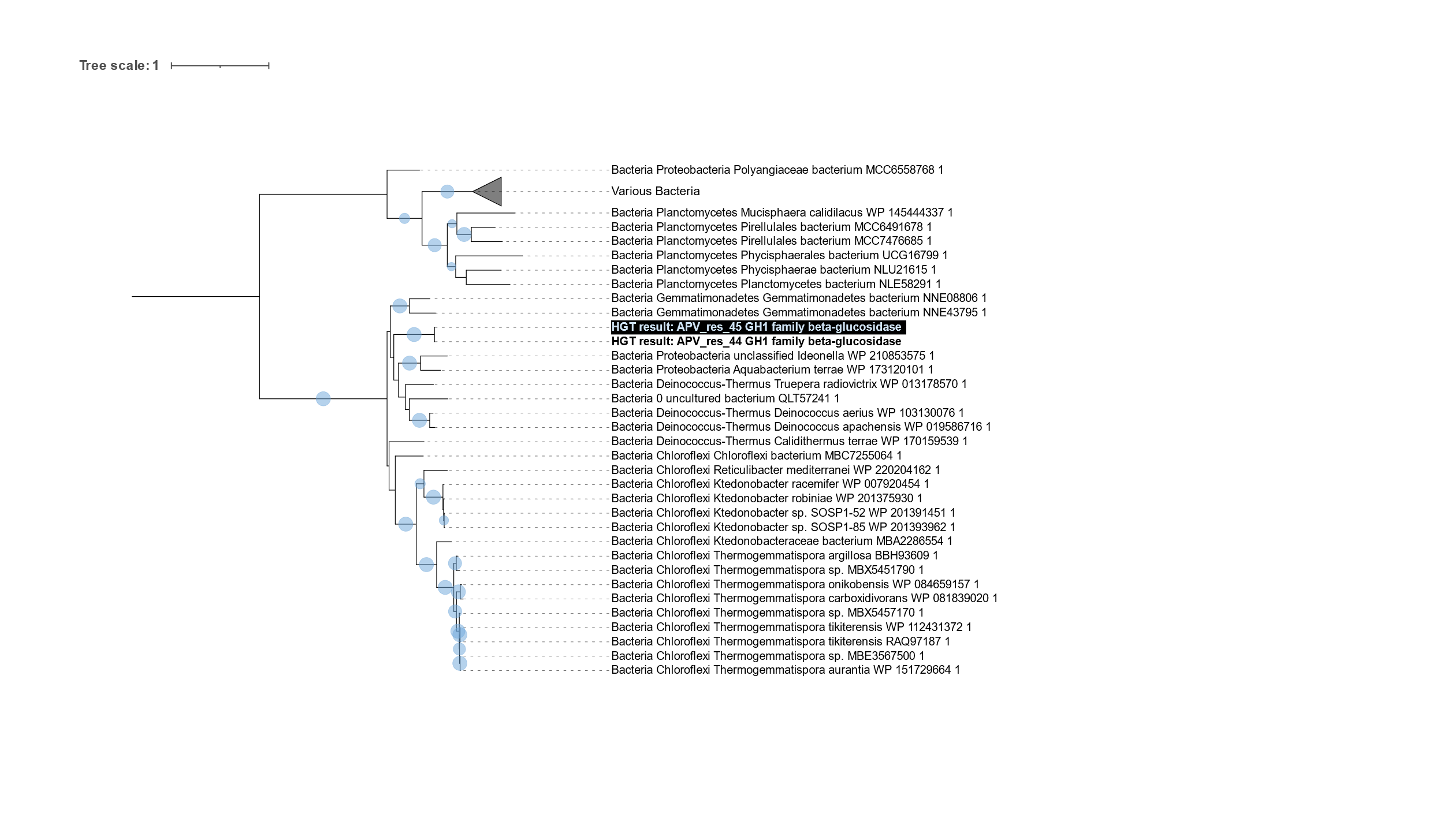


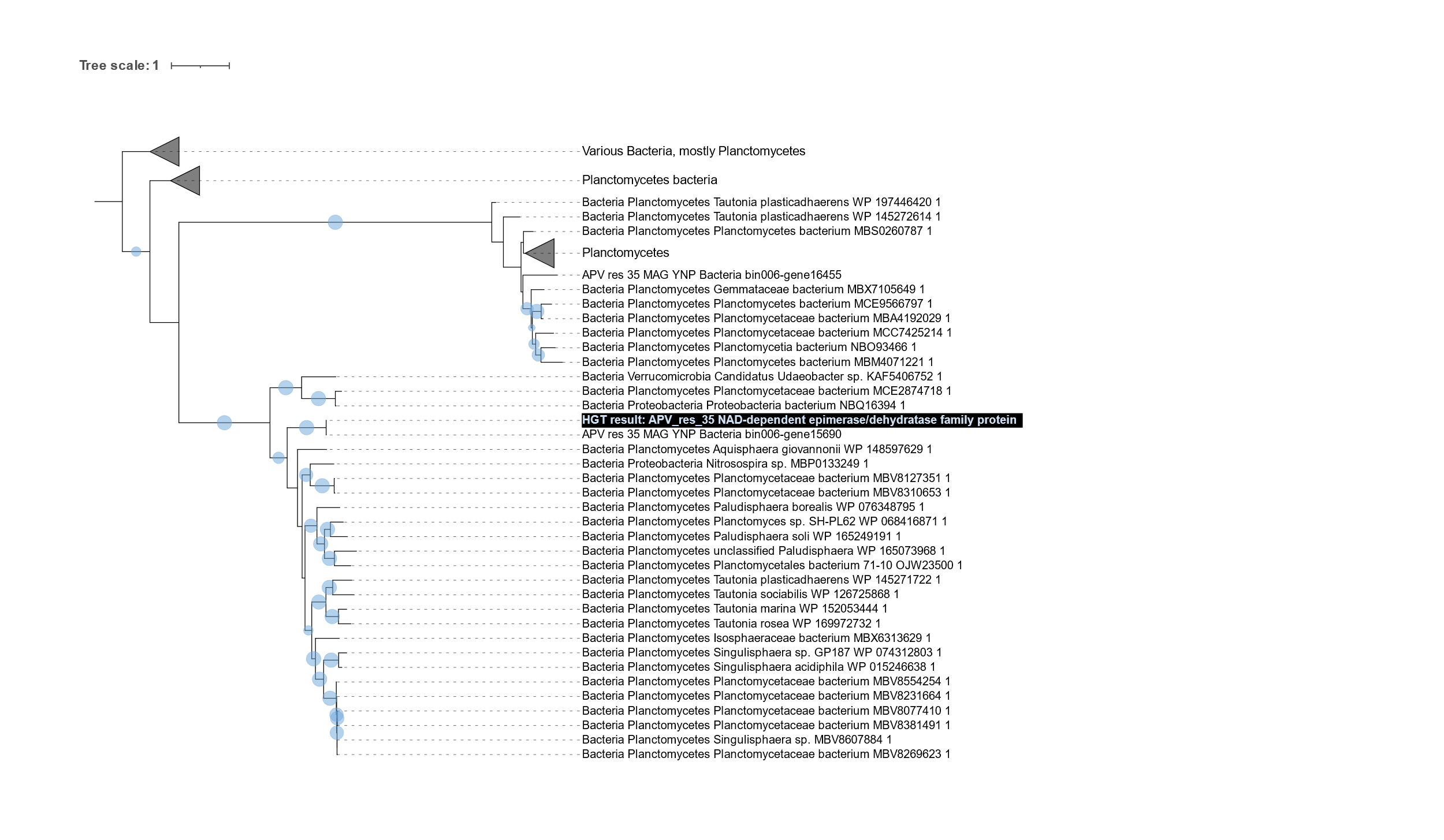


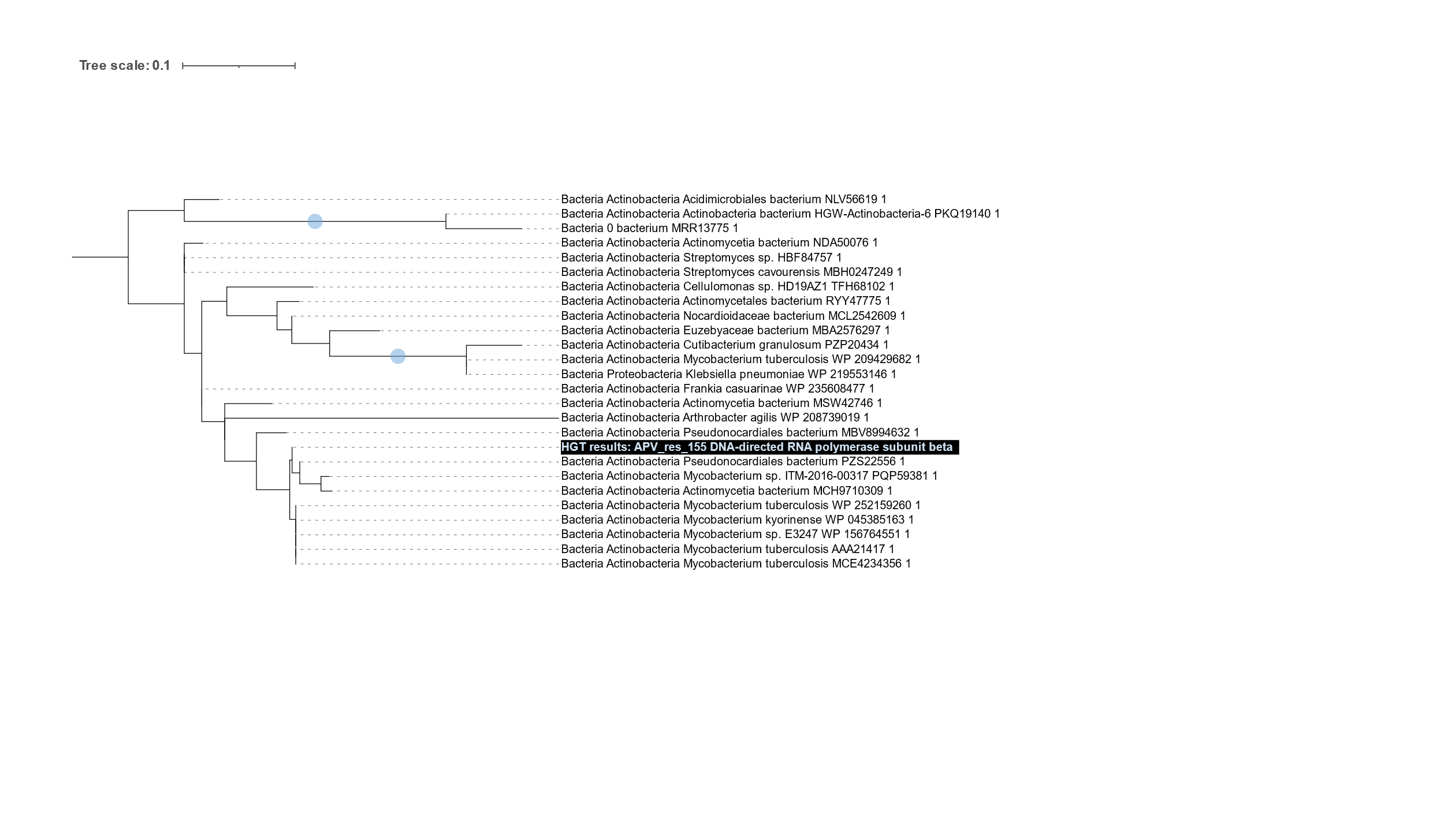


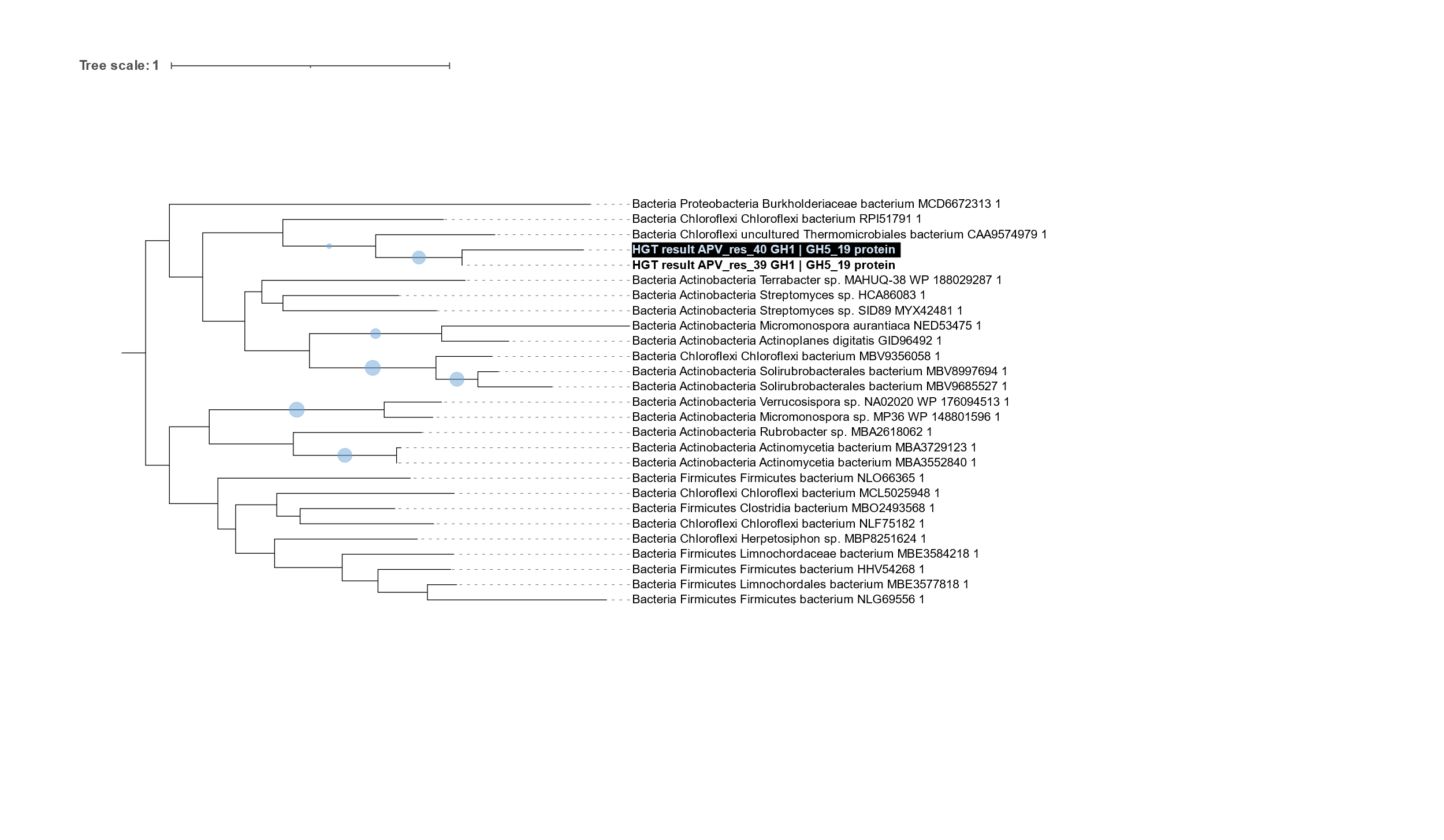

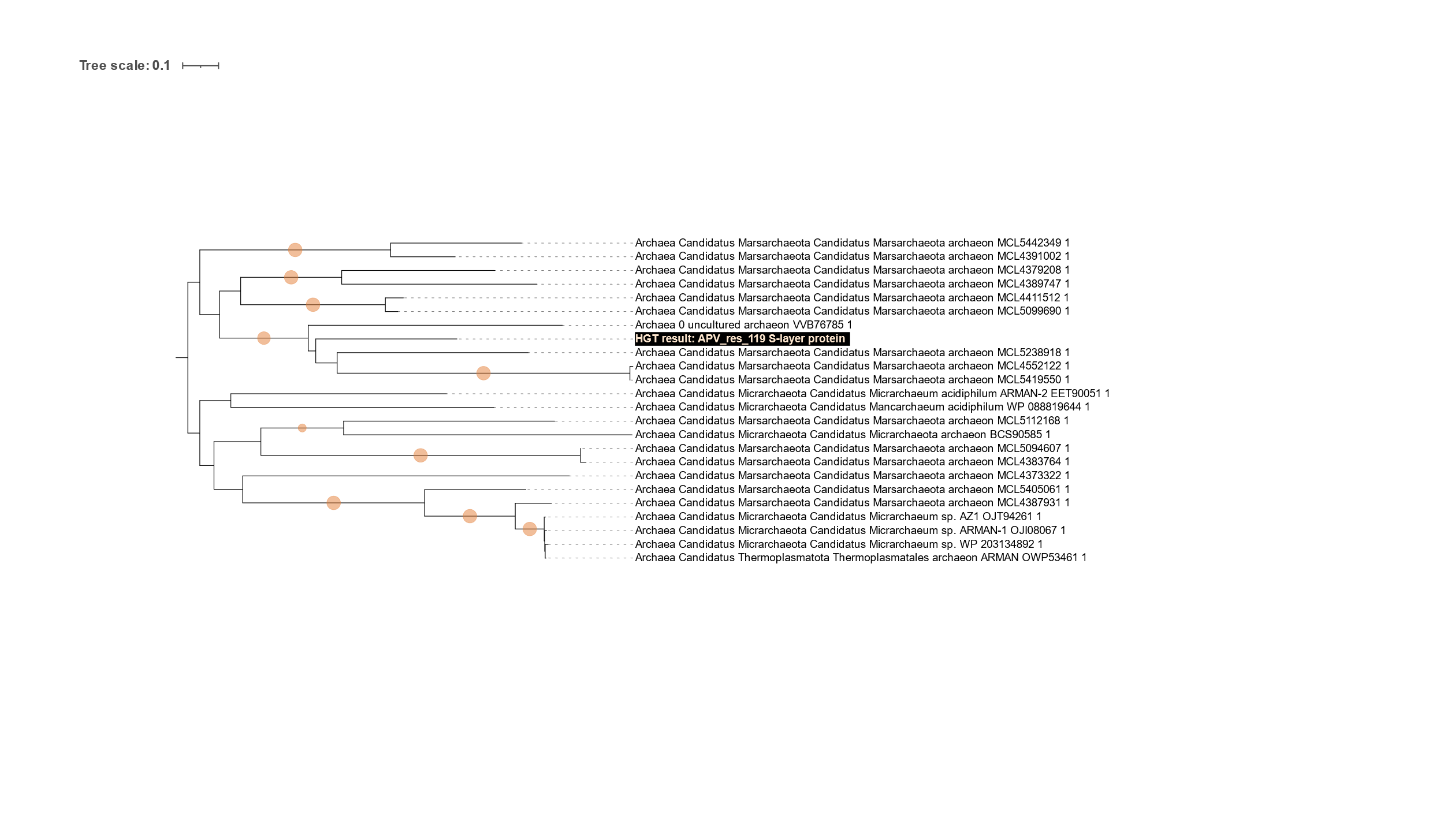


**Figure S2.** This figure is a composite of all single-gene amino acid phylogenies lacking a clear functional annotation. Below this figure are larger versions of all 6 gene trees. Circles are bootstrap values between 90 and 100. All trees are midpoint rooted.
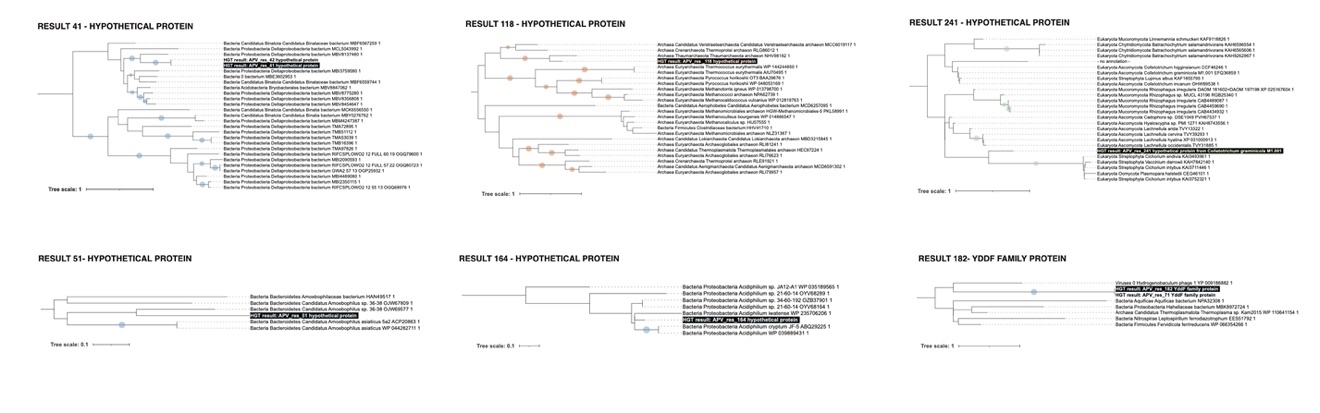


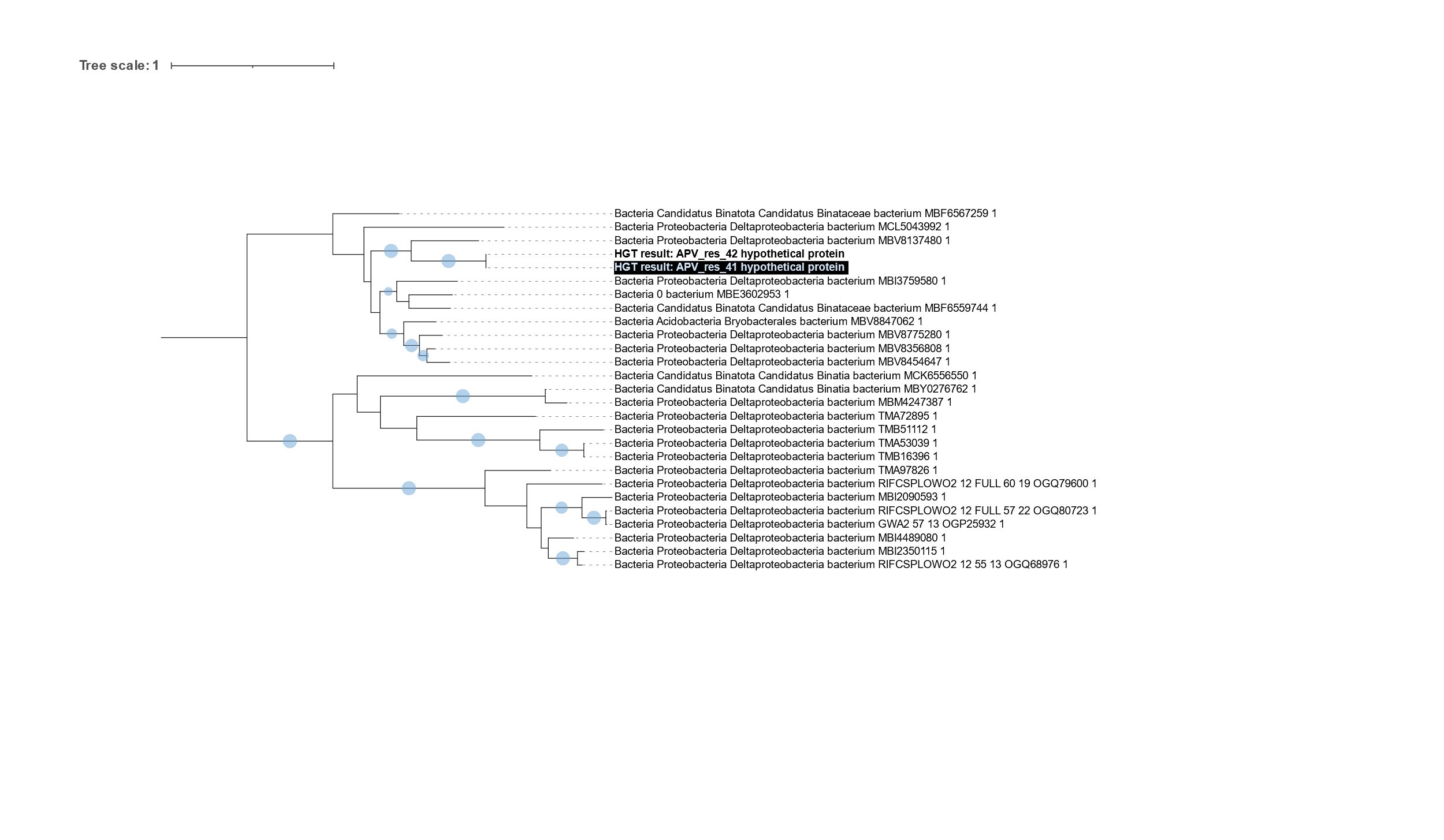


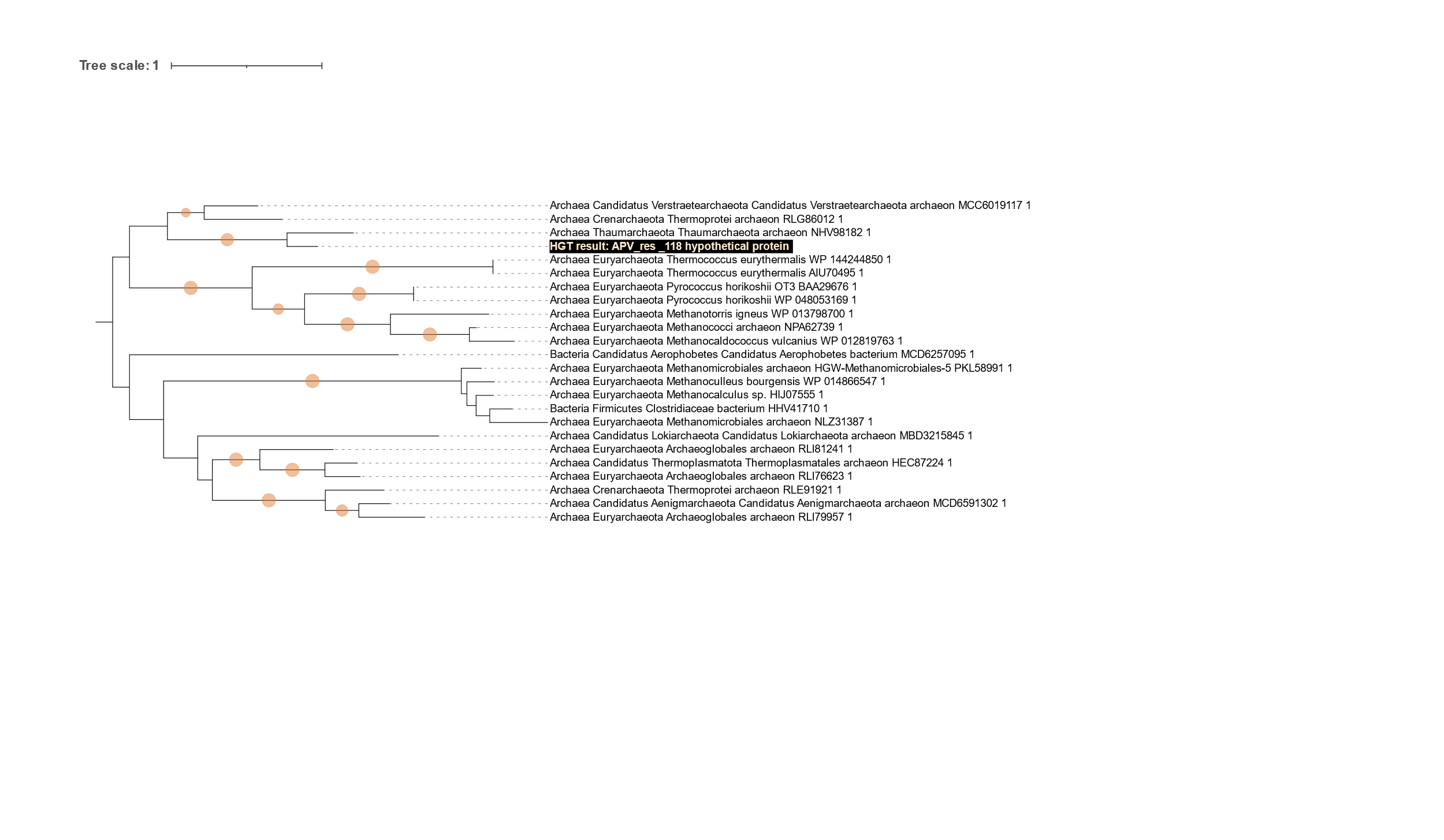

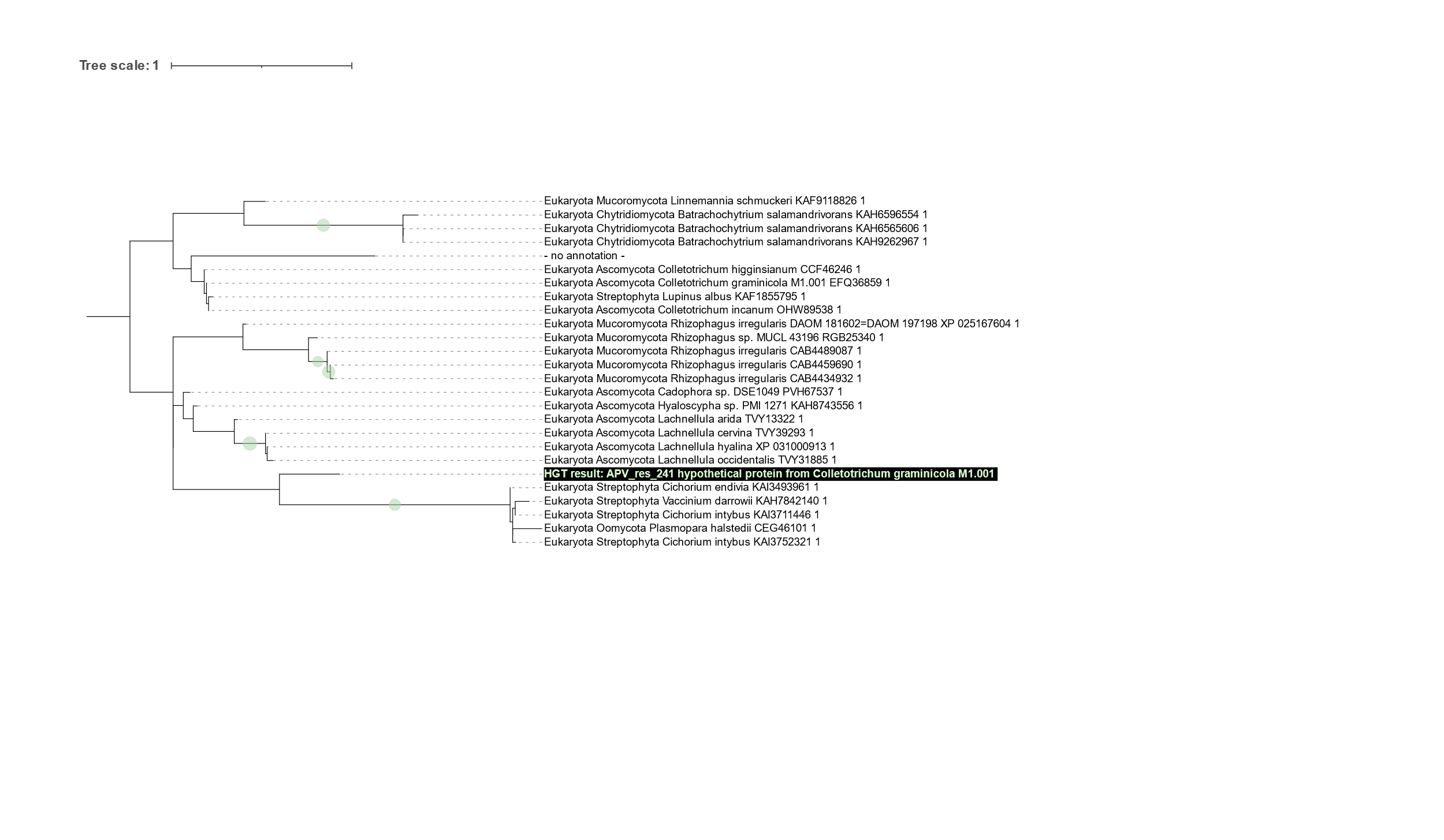

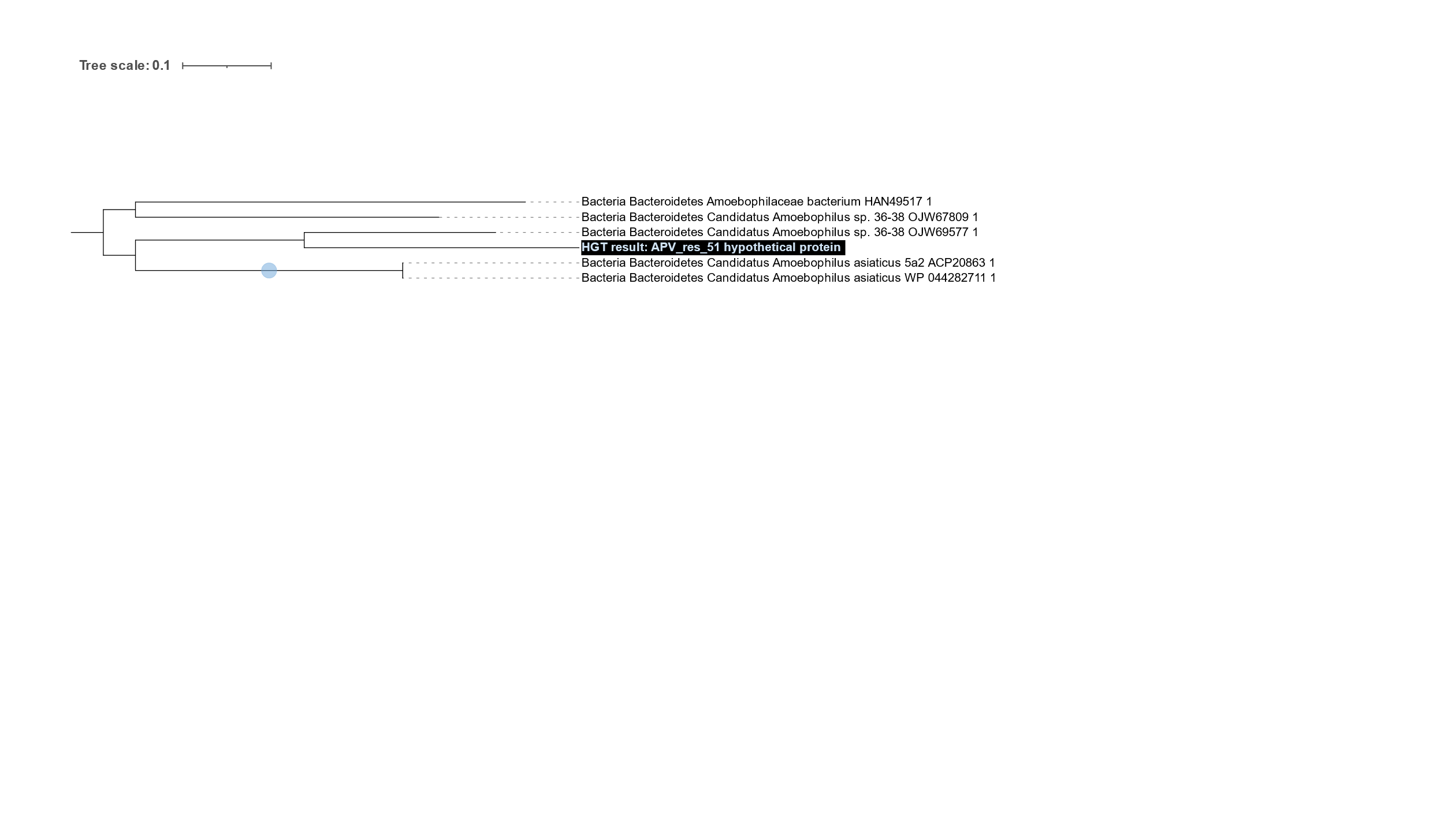

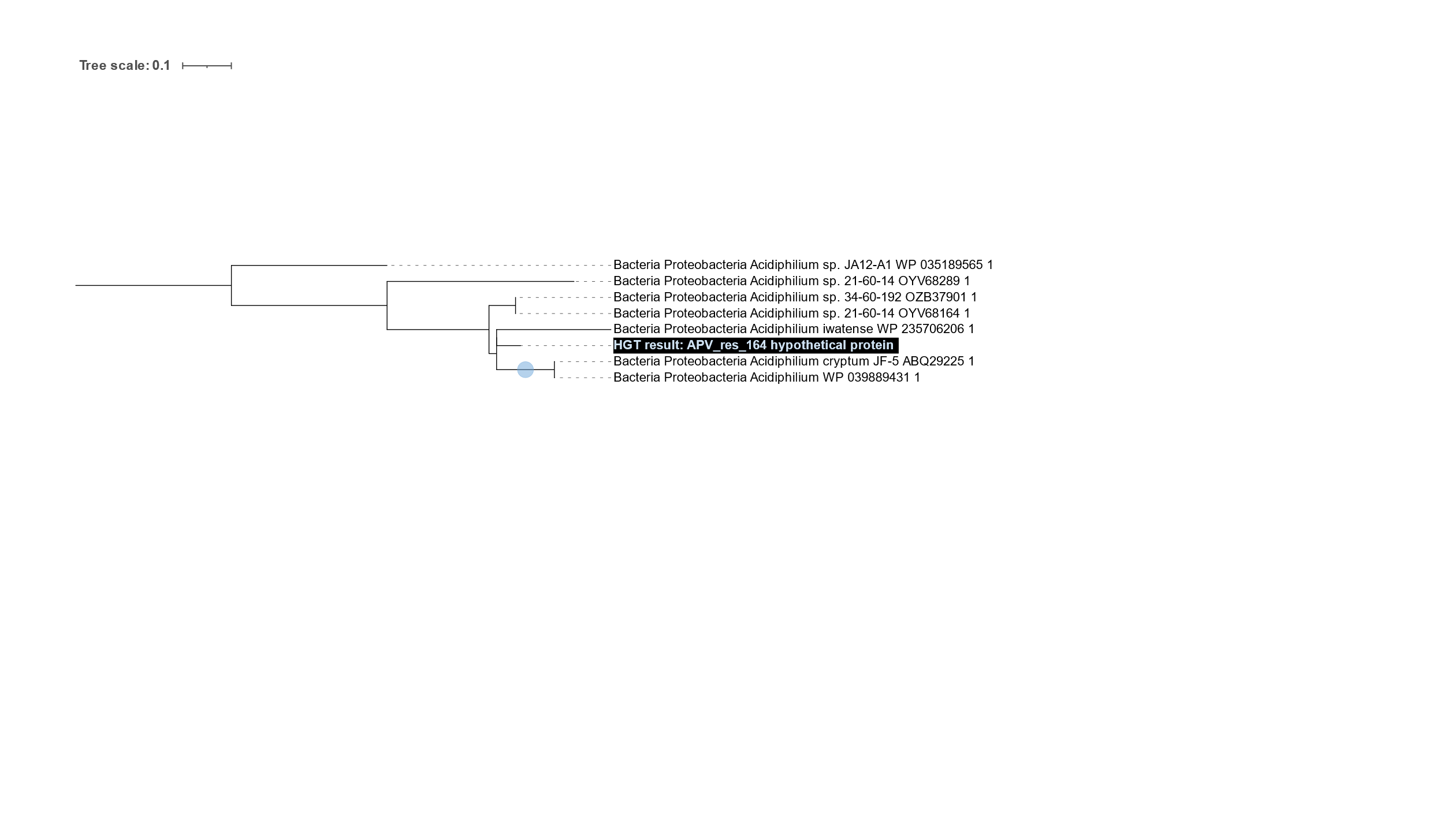

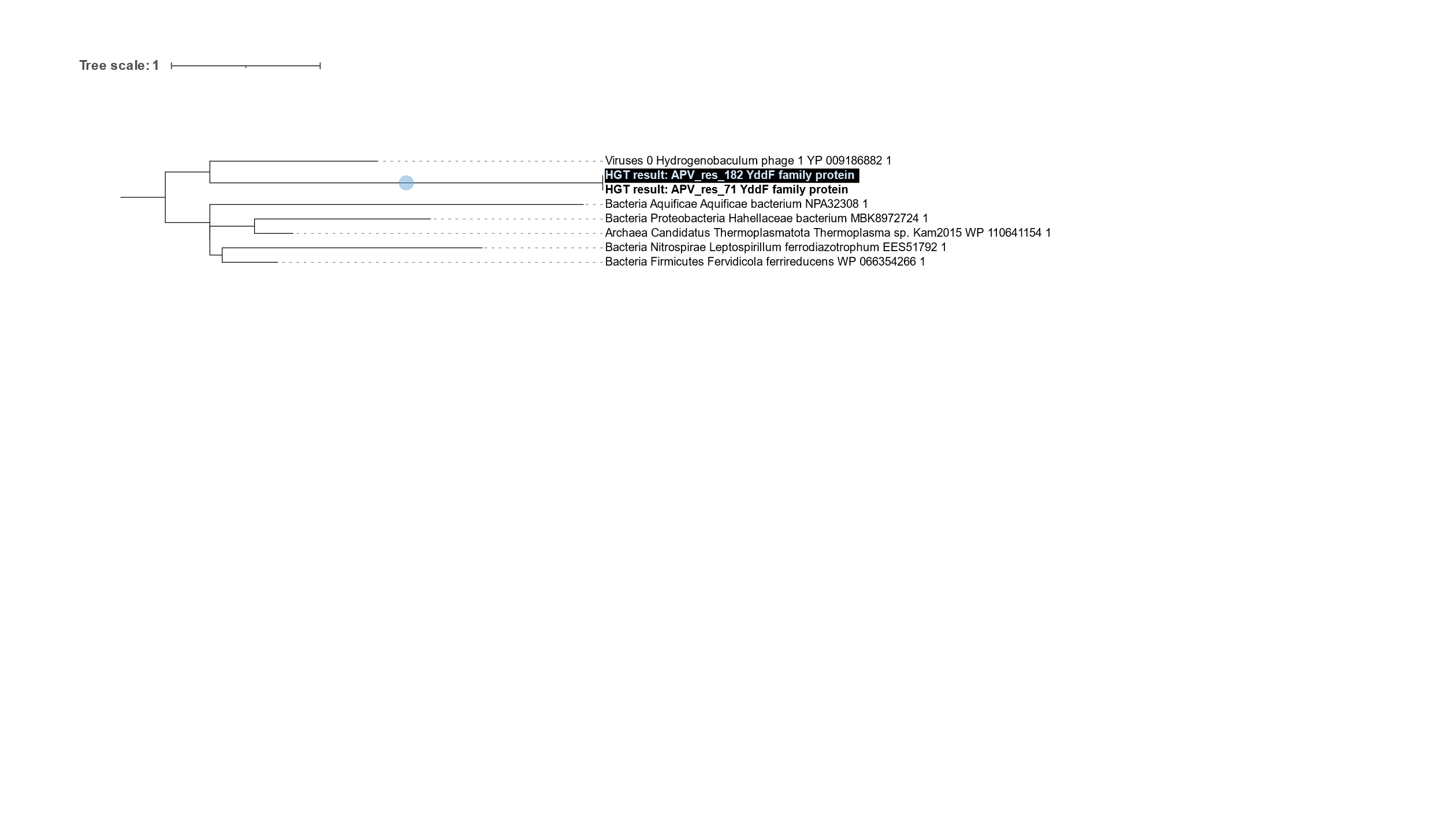


**Table S1.** For access to Table S1 which shows raw results, filtering statistics, and where in the pipeline each result got filtered out, visit the Zenodo repository: doi.org/10.5281/zenodo.10529606

2. Supplementary Table S1

**Table S2.** For access to Table S2 which shows assembly statistics for the two Cyanidiophyceaen MAGs, visit the Zenodo repository: doi.org/10.5281/zenodo.10529606

3. Supplementary Table S2

**Table S3.** For access to Table S2 which shows assembly statistics for the prokaryotic MAGs, visit the Zenodo repository: doi.org/10.5281/zenodo.10529606

4. Supplementary Table S3

**Table S4.** The MAGs and vOTUs used in each class categorization from which putative HGT results were derived. Note: the two algae are separate genera within the same class but were kept as separate for this analysis. MAG and vOTU .fasta files can be found at <https://www.biorxiv.org/content/10.1101/2024.09.03.611078v1> and <https://zenodo.org/doi/10.5281/zenodo.12710562>. A = algae, P(b) = bacteria, P(a) = archaea, V = viruses.

| **APV** | **Taxonomic class** | **MAGs included (bin #)** | **# MAGs/ vOTUs** |
| --- | --- | --- | --- |
| A | Genus: *Galdieria* | *G. yellowstonensis* YNP5587.1 | 1 |
| A | Genus: *Cyanidioschyzon* | *C. merolae* 10D | 1 |
| P(b) | Acidimicrobiia | b013, b021, b024, b025, b026, b027, b028, b029, b030, b031, b033, b034, b035, b037, b039, b040, b042, b044, b045, b048, b056, b058, b060, b062, b063, b064, b065, b068, b069, b070, b071, b074, b075, b076, b078, b079, b080, b082, b086, b088, b092, b094, b095, b098, b099, b104, b015, b109, b114, b125, b127, b129, b131, b133 | 54 |
| P(b) | Actinomnycetia | b002, b004, b005, b006, b009 | 5 |
| P(b) | Alphaproteobacteria | b012, b014, b015, b018, b020, b041, b072, b083, b097, b101, b102, b107, b110, b119, b120, b123, b136 | 17 |
| P(b) | Aquificae | b089, b103 | 2 |
| P(b) | Babeliae | b116, b117 | 2 |
| P(b) | Bacilli | b023, b047, b049, b052, b111 | 5 |
| P(b) | Bacteroidia | b085, b137 | 2 |
| P(b) | Binatia | b008, b016, b073, b081 | 4 |
| P(b) | Chlamydiia | b057 | 1 |
| P(b) | Deinococci | b053 | 1 |
| P(b) | Desulfotomaculia | b046 | 1 |
| P(b) | Desulfurellia | b096 | 1 |
| P(b) | Dormibacteria | b038, b043 | 2 |
| P(b) | Gammaproteobacteria | b019, b022, b036, b055, b059, b067, b091, b134, b139 | 9 |
| P(b) | Leptospirillia | b050, b051, b061 | 3 |
| P(a) | Micrarchaeia | a029, a032, a033, a034, a035, a036, | 6 |
| P(a) | Nanoarchaeia | a031 | 1 |
| P(a) | Nitrososphaeria | a009, a015, a020 | 3 |
| P(b) | Phycisphaerae | b010 | 1 |
| P(b) | Planctomycetia | b001, b003, b007, b093 | 4 |
| P(b) | RBS10-35 | b100, b115 | 2 |
| P(b) | Saccharimonadia | b108, b113, b121, b122, b124, b126 | 6 |
| P(b) | Sulfobacillia | b017, b054, b066, b112 | 4 |
| P(b) | SZUA-79 | b084, b087 | 2 |
| P(b) | Thermoleophilia | b011, b032, b106, b118 | 4 |
| P(a) | Thermoplasmata | a003, a004, a005, a006, a007, a008, a010, a011, a012, a013, a014, a015, a016, a017, a018, a019, a022, a023, a024, a027, a028 | 20 |
| P(a) | Thermoproteia | a001, a002, a021, a025, a026, a030 | 6 |
| P(b) | Thermotogae | b007, b090 | 2 |
| V | Cressdnaviricota | bin.1029, bin.941, bin.1041, bin.569, bin.594, bin.690, bin.901 | 7 |
| V | Nucleoviricota | bin.750, bin.932 | 2 |
| V | Unknown dsDNA virus, family Pithoviridae | bin.1304, bin.376, bin.403, bin.421, bin.757, bin.763, bin.979 | 7 |
| V | Pandoravirus | bin.800 | 1 |
| V | Preplasmviricota | bin.250 | 1 |
| V | Unknown106 | bin.130 | 1 |
| V | Unknown107 | bin.193 | 1 |
| V | Unknown 108 | bin.232 | 1 |
| V | Unknown109 | bin.705 | 1 |
| V | Unknown110 | bin.787 | 1 |
| V | Unknown111 | bin.795 | 1 |
| V | Unknown112 | bin.871 | 1 |

**Supplemental files.** Newick tree files used in this analysis (to make Figures 3, S1, and S2) can be found in the Zenodo repository for this project here: doi.org/10.5281/zenodo.10529606. Note: after initial drafting of this manuscript, Cyanidiophyceae taxonomy was updated (changes reflected in the main text) so any supplemental files, including trees and spreadsheets may use the old species name *Galdieria sulphuraria.* The updated name for this organism is *Galdieria yellowstonensis.*

5. 1_Rendonuclease_treeinput.fasta.mafft.contree.tax

6. 1_res110MTase_treeinput.fasta.mafft.contree.tax

7. 1_res118_treeinput.fasta.mafft.contree.tax

8. 1_res119_treeinput.fasta.mafft.contree.tax

9. 1_res144_treeinput.fasta.mafft.contree.tax

10. 1_res155_treeinput.fasta.mafft.contree.tax

11. 1_res164_treeinput.fasta.mafft.contree.tax

12. 1_res241_treeinput.fasta.mafft.contree.tax

13. 1_res35_treeinput.fasta.mafft.contree.tax

14. 1_res3940_treeinput.fasta.mafft.contree.tax

15. 1_res4142_treeinput.fasta.mafft.contree.tax

16. 1_res4445_treeinput.fasta.mafft.contree.tax

17. 1_res51_treeinput.fasta.mafft.contree.tax

18. 1_res5354_treeinput.fasta.mafft.contree.tax

19. 1_res71182_treeinput.fasta.mafft.contree.tax

20. 1_res76_treeinput.fasta.mafft.contree.tax

21. 1_transposase_treeinput.fasta.mafft.contree.tax
